# Supplementary material for: CREB1-driven CXCR4hi neutrophils promote skin inflammation in mouse models and human patients
Source: Nat Commun. 2023 Sep 22;14:5894. doi: 10.1038/s41467-023-41484-3 (PMC10516899; doi:10.1038/s41467-023-41484-3)
Supplement: Supplementary file 1 — Supplementary Information [file 41467_2023_41484_MOESM1_ESM.pdf]

Supplementary Information for

CREB1-driven CXCR4^{hi} neutrophils promote skin inflammation in mouse models and human patients

Jiaoling Chen[1,#], Yaxing Bai[1,#], Ke Xue[1], Zhiguo Li[1], Zhenlai Zhu[1], Qingyang Li[1], Chen Yu[1], Bing Li[1], Shengxian Shen[1], Pei Qiao[1], Caixia Li[1], Yixin Luo[1], Hongjiang Qiao[1], Erle Dang[1], Wen Yin[2], Johann E. Gudjonsson[3,*], Gang Wang[1,*], Shuai Shao[1,*]

Affiliations:

[1] Department of Dermatology, Xijing Hospital, Fourth Military Medical University, Xi'an, Shaanxi 710032, China

[2] Department of Transfusion Medicine, Xijing Hospital, Fourth Military Medical University, Xi'an, Shaanxi 710032, China

[3] Department of Dermatology, University of Michigan, Ann Arbor, Michigan 48109, USA

[#] Equally contributing authors

This file includes:

Supplementary Figures:

Supplementary Fig. 1. Predominance of $CXCR4^{hi}$ neutrophils in psoriasis.

Supplementary Fig. 2. Hyperactivation of psoriatic $CXCR4^{hi}$ neutrophils.

Supplementary Fig. 3. The NETs formation and phagocytosis capacity of $CXCR4^{hi}$ neutrophils.

Supplementary Fig. 4. $CXCR4^{hi}$ neutrophils show higher glycolysis levels than $CXCR4^{lo}$ neutrophils.

Supplementary Fig. 5. Psoriatic $CXCR4^{hi}$ neutrophils downregulate the expression of tight junctions in HMEC-1 cells.

Supplementary Fig. 6. Psoriatic $CXCR4^{hi}$ neutrophils regulate vascular remodeling via the lactate-GPR81 pathway.

Supplementary Fig. 7. CXCL12 is mainly derived from vascular endothelium and fibroblasts in psoriatic lesions.

Supplementary Fig. 8. CXCR4 expression on neutrophils is not regulated by degranulation.

Supplementary Fig. 9. CREB1 promotes the development of $CXCR4^{hi}$ neutrophils.

Supplementary Fig. 10. Verification of CREB1 inhibition efficiency.

Supplementary Fig. 11. Blocking CREB1 impairs the pro-inflammatory functions of psoriatic $CXCR4^{hi}$ neutrophils.

Supplementary Fig. 12. Analysis for the efficiency of neutrophil depletion and infiltration of immune cells in mouse skin, related to Fig. 7.

Supplementary Fig. 13. Validation of AMD3100 inhibition efficiency at various concentrations.

Supplementary Fig. 14. Validation of anti-CXCL12 inhibition efficiency at various concentrations.

Supplementary Fig. 15. CXCL12 regulates $CXCR4^{hi}$ neutrophils in psoriasis-like mouse model.

Supplementary Fig. 16. The frequency of $CD177^{+}$ and $OLFM4^{+}$ neutrophils in healthy controls and psoriasis patients.

Supplementary Fig. 17. The isolation efficiency of separated $CXCR4^{hi}$ neutrophils via magnetic method.

Supplementary Tables:

Supplementary Table 1. Patient characteristics.

Supplementary Table 2. Sequences of primers for real-time PCR.

Supplementary Figures

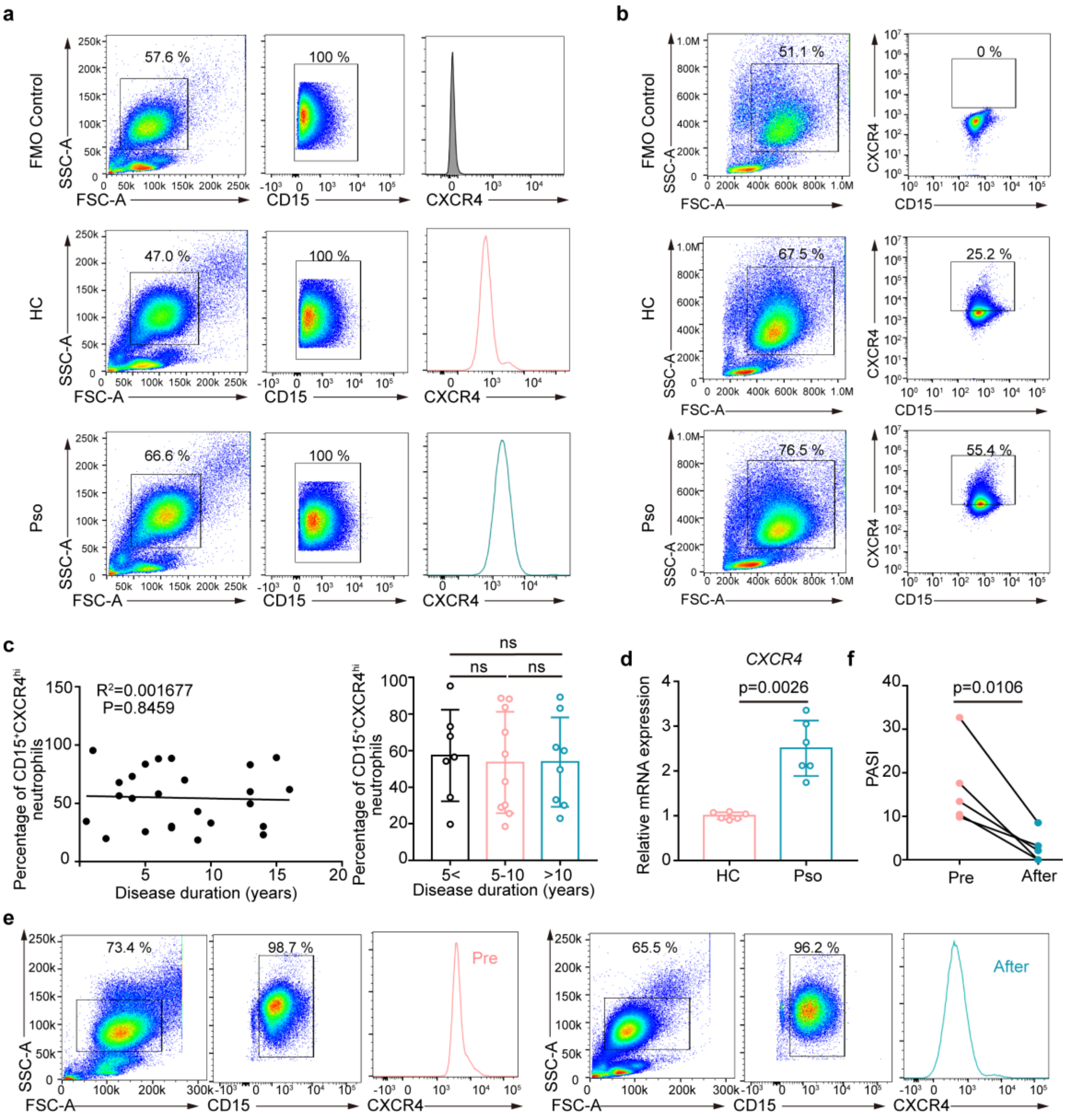

Supplementary Fig. 1. Predominance of CXCR4^{hi} neutrophils in psoriasis. (**a, b**) Gating strategy for CXCR4 fluorescence intensity on peripheral neutrophils (**a**) and proportion of CXCR4^{hi} neutrophils (**b**) in healthy controls and psoriasis patients, related to Fig. 1a and 1c. Analysis performed on whole blood. (**c**) Correlation of the percentage of psoriatic CXCR4^{hi} neutrophils with disease durations (n = 25). (**d**) Relative mRNA expression of CXCR4 in neutrophils from healthy controls and psoriasis patients (n = 6). (**e**) Gating strategy for the fluorescence intensity of CXCR4 on neutrophils from psoriasis patients before and after treatment with anti-IL-17 inhibitor for 12 weeks, related to Fig. 1h. Analysis performed on whole blood. (**f**) PASI score before and after 12 weeks of treatment with anti-IL-17 inhibitor in psoriasis patients (n = 5). Data are mean ± SD. Analyses: The Spearman method and one-way ANOVA with Tukey's post hoc test in **c**; Unpaired Student's t-test in **d**; Paired Student's t-test in **f**. The unpaired

Student's t-test was conducted as two-sided tests. One-way ANOVA test was performed as two-sided analyses and adjusted for multiple comparisons in the statistical analyses. ns, no significance. HC, healthy control; MFI, mean fluorescence intensity; PASI, psoriasis area and severity index; Pre, pre-treatment; Pso, psoriasis patients. Source data are provided as a Source Data file.

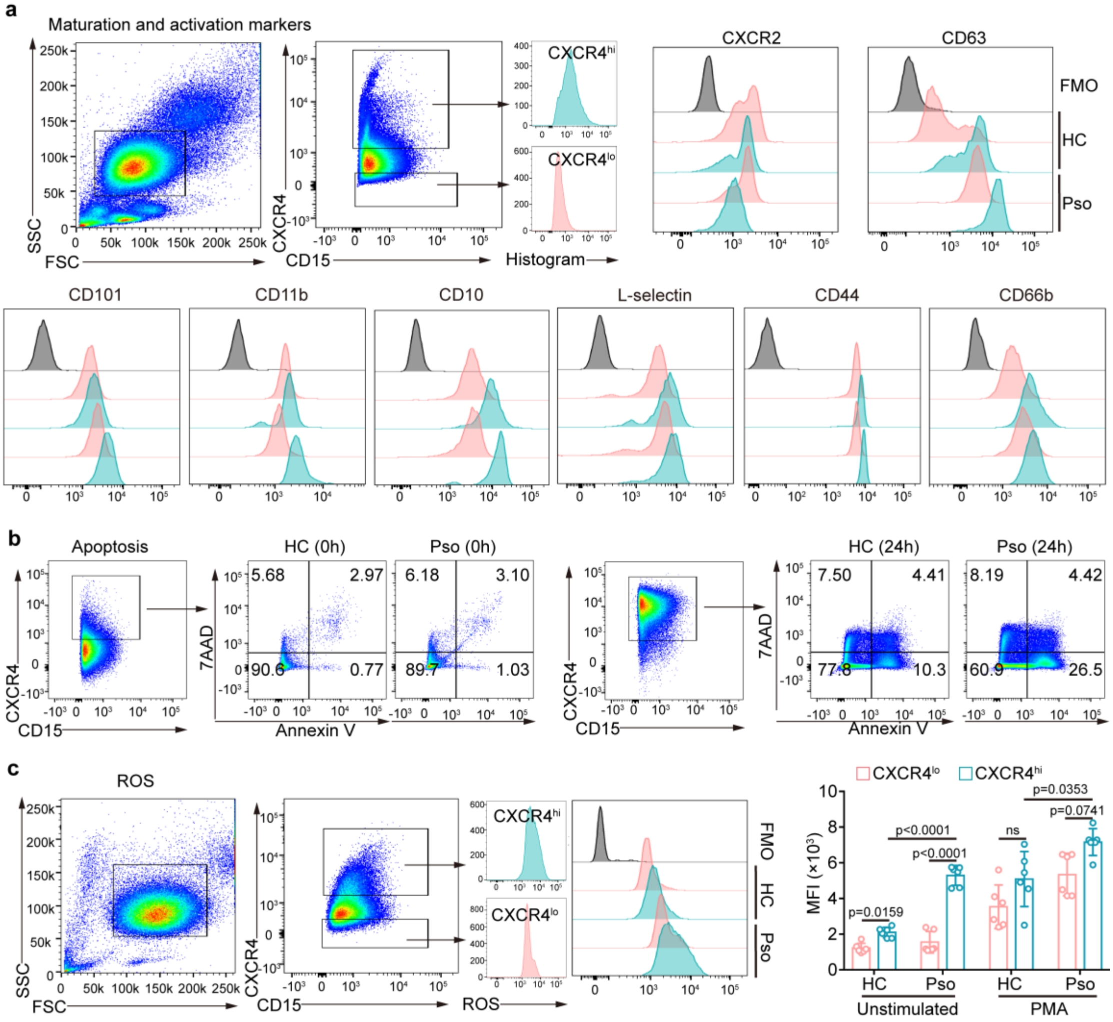

Supplementary Fig. 2. Hyperactivation of psoriatic CXCR4^{hi} neutrophils. (**a**) Gating strategy and representative flow cytometry histogram of key immune markers between peripheral CXCR4^{lo} and CXCR4^{hi} neutrophils from healthy controls and psoriasis patients, related to Fig. 2b. Whole blood was used for analysis. (**b**) Gating strategy and data for early/late apoptosis of CXCR4hi neutrophils cultured for 24 hours, related to Fig. 2c. (**c**) Gating strategy and data for ROS level detected in CXCR4^{lo} and CXCR4^{hi} neutrophils from healthy controls and psoriasis patients, related to Fig. 2d. Isolated neutrophils from peripheral blood were used for analysis. Data are mean ± SD (n = 6 biologically independent samples). Analyses: two-way ANOVA with Tukey's post hoc test was used in **c**. Two-way ANOVA test was performed as two-sided analyses and adjusted for multiple comparisons in the statistical analyses. ns, not significant. FMO, Fluorescence Minus One; HC, healthy control; MFI, mean fluorescence intensity; PMA, Phorbol 12-myristate 13-acetate; Pso, psoriasis patients. Source data are provided as a Source Data file.

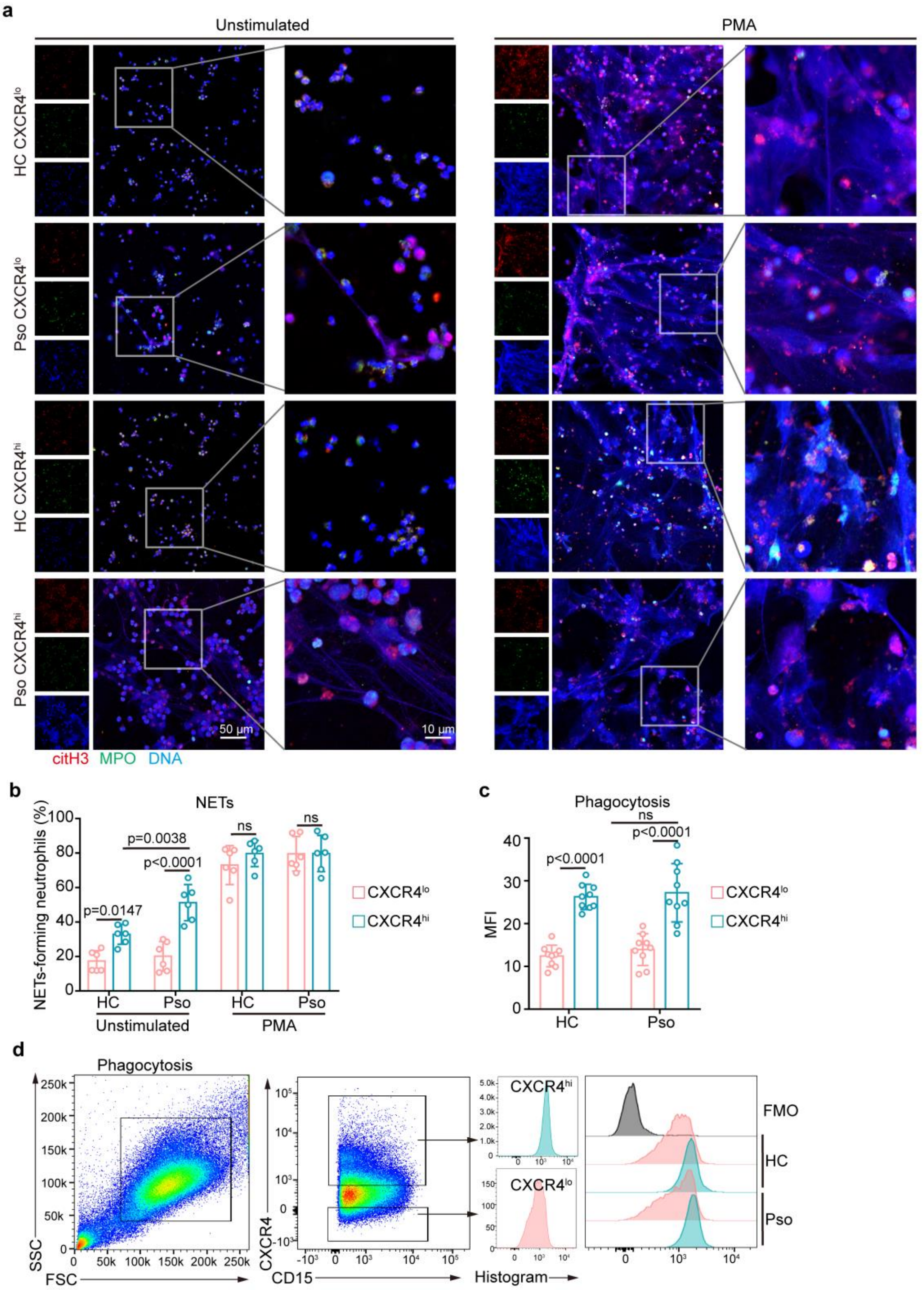

Supplementary Fig. 3. The NETs formation and phagocytosis capacity of CXCR4^{hi} neutrophils. (**a**) Representative immunofluorescence co-staining of DNA (Hoechst), Cit-H3 (citrullinated histone-3, red), and MPO (myeloperoxidase, green) to assess NETs formation in CXCR4^{lo} and CXCR4^{hi} neutrophils isolated from peripheral blood of healthy controls and psoriasis patients with/without PMA induction. Scale bar = 50

μm, 10 μm. (**b**) Quantification of NETs in CXCR4^{lo} and CXCR4^{hi} neutrophils from healthy controls and psoriasis patients with/without PMA induction. (**c**) Quantification for phagocytosis based on pHrodo Green *E. coli* immunofluorescence staining, related to Fig. 2f. (**d**) Gating strategy for the phagocytosis of peripheral CXCR4^{lo} and CXCR4^{hi} neutrophils from healthy controls and psoriasis patients, related to Fig. 2g. Isolated neutrophils from peripheral blood were used. Data are mean ± SD (n = 6 biologically independent samples/group). The immunofluorescence staining was repeated three times independently with similar results. Analyses: two-way ANOVA with Tukey's post hoc test. Two-way ANOVA test was performed as two-sided analyses and adjusted for multiple comparisons in the statistical analyses. ns, not significant. citH3, Citrullinated Histone H3; FMO, Fluorescence Minus One; HC, healthy control; MFI, mean fluorescence intensity; NETs; neutrophil extracellular traps; PMA, Phorbol 12-myristate 13-acetate; Pso, psoriasis patients. Source data are provided as a Source Data file.

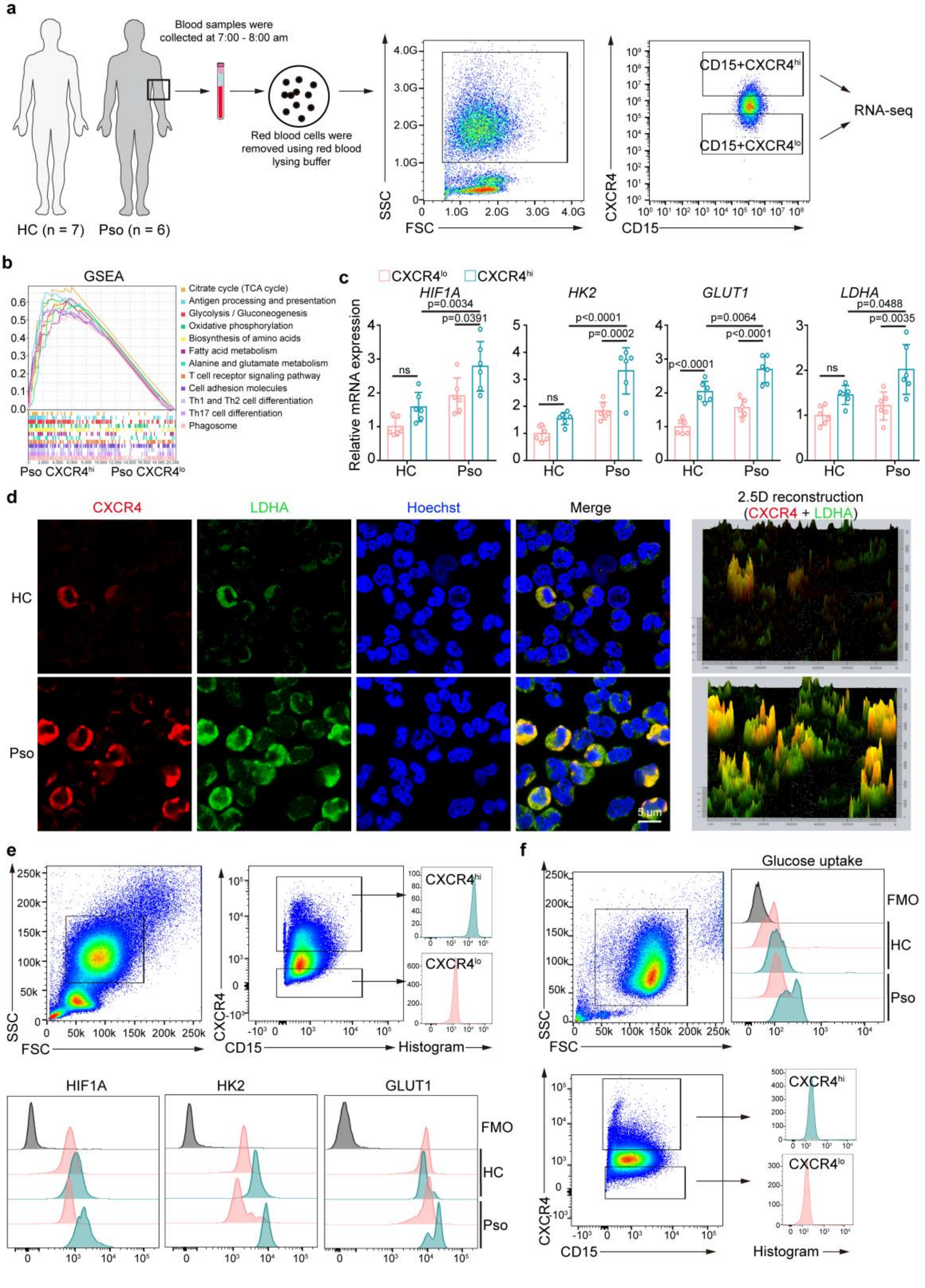

Supplementary Fig. 4. CXCR4^{hi} neutrophils show higher glycolysis levels than CXCR4^{lo} neutrophils. (**a**) Schematic diagram of isolating CXCR4^{lo} and CXCR4^{hi} neutrophils from psoriasis patients (n = 6) and sex-, age-matched healthy controls (n = 7) for bulk RNA-seq. 20 mL whole blood was collected from each individual at 7:00 - 8:00 am and red blood cells were removed using red blood lysing buffer. Total blood

leukocytes were incubated with FITC conjugated anti-human CD15 and PE-Cy7 conjugated anti-human CXCR4 for isolating $CXCR4^{lo}$ and $CXCR4^{hi}$ neutrophils by flow cytometry. 1×10^6 $CXCR4^{lo}$ and $CXCR4^{hi}$ neutrophils were collected for bulk RNA-seq. (**b**) Related pathway analysis between $CXCR4^{hi}$ and $CXCR4^{lo}$ neutrophils from psoriasis patients was carried out through gene set enrichment analysis, related to Fig. 3c. (**c**) Relative mRNA expression of glycolytic genes in $CXCR4^{lo}$ and $CXCR4^{hi}$ neutrophils from healthy controls and psoriasis patients. (**d**) Representative immunofluorescence co-localization of CXCR4 (red) and LDHA (green) in neutrophils from healthy controls and psoriasis patients, and 2.5D reconstruction with overlay was established. Scale bar = 5 µm. (**e**) Gating strategy for glycolytic markers in $CXCR4^{lo}$ and $CXCR4^{hi}$ neutrophils from healthy controls and psoriasis patients, related to Fig. 3e. (**f**) Gating strategy for glucose uptake, related to Fig. 3f. Whole blood was used for **e** and isolated neutrophils from peripheral blood were used for **f**. Mean ± SD (n = 6 biologically independent samples). The immunofluorescence staining was repeated three times independently with similar results. Two-way ANOVA with Tukey's post hoc test in **b** was performed as two-sided analyses and adjusted for multiple comparisons in the statistical analyses. ns, not significant. FMO, Fluorescence Minus One; HC, healthy control; Pso, psoriasis patients. Source data are provided as a Source Data file.

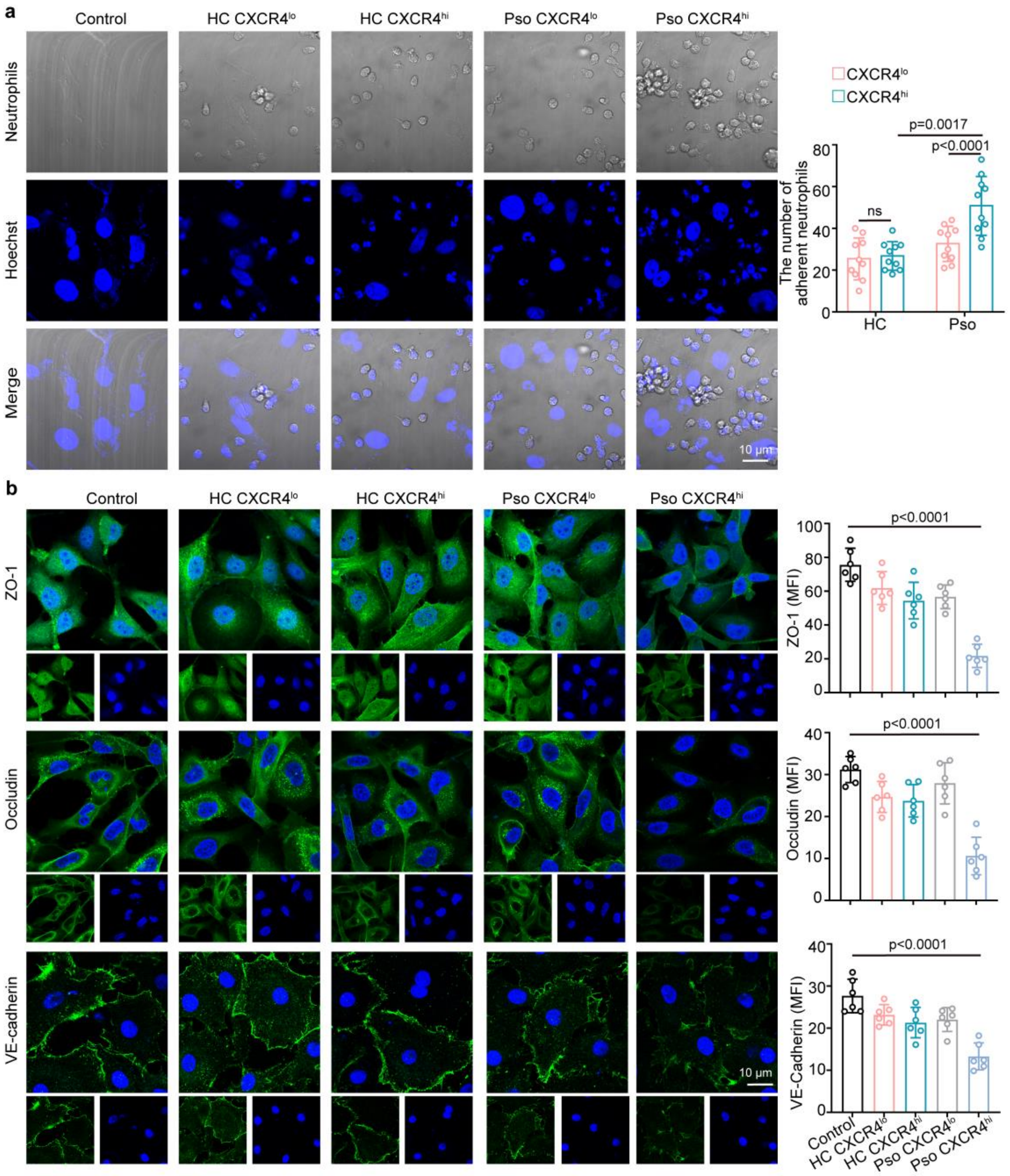

Supplementary Fig. 5. Psoriatic CXCR4hi neutrophils downregulate the expression of tight junctions in HMEC-1 cells. (**a**) Representative images and quantification of adherent CXCR4lo and CXCR4hi neutrophils co-cultured with HMEC-1 cells. (**b**) Immunofluorescence staining and quantification of MFI in HMEC-1 cells with indicated treatment. Scale bar = 10 µm. Mean ± SD (n = 6 biologically independent samples/group). The immunofluorescence staining was repeated three times independently with similar results. Analyses: two-way ANOVA with Tukey's post hoc test in **a**; One-way ANOVA with Tukey's post hoc test in **b**. One or two-way ANOVA tests were performed as two-sided analyses and adjusted for multiple comparisons in the statistical analyses. ns, not significant. HC, healthy control; MFI, mean fluorescence intensity; Pso, psoriasis patients. Source data are provided as a Source Data file.

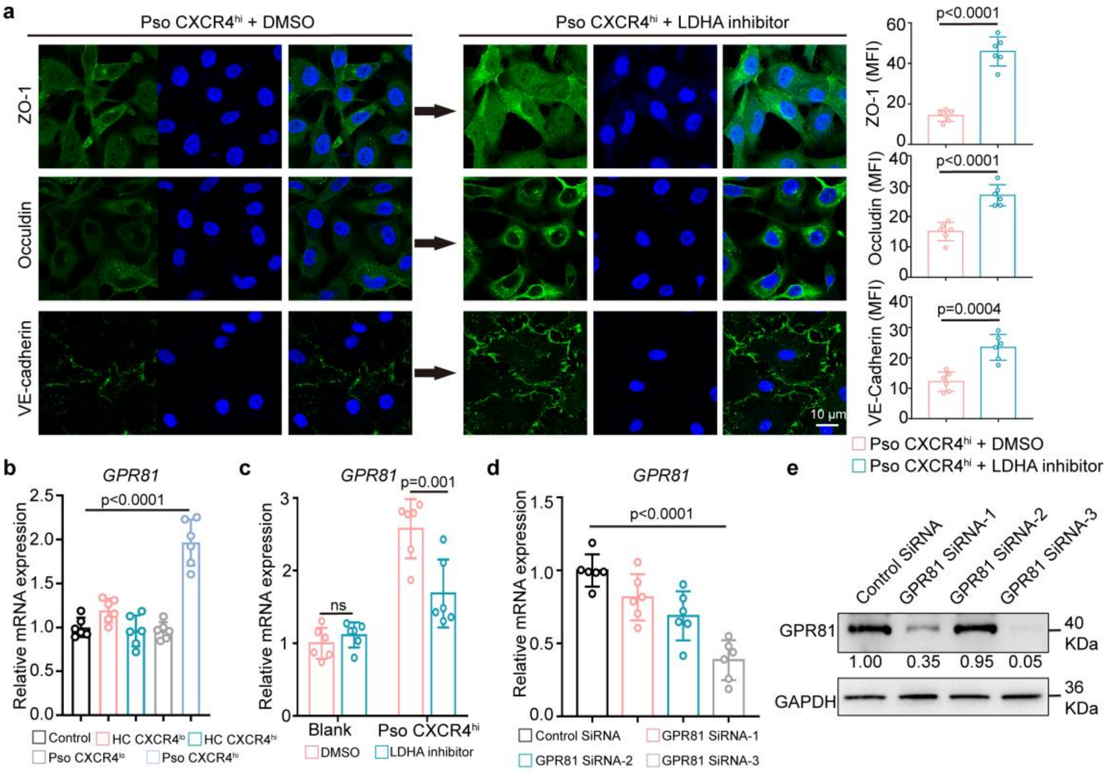

Supplementary Fig. 6. Psoriatic CXCR4^{hi} neutrophils regulate vascular remodeling via the lactate-GPR81 pathway. (**a**) Immunofluorescence analysis of HMEC-1 cells pre-incubated with LDHA inhibitor for 30 min following co-culture with psoriatic CXCR4^{hi} neutrophils and MFI was quantified. Scale bar = 10 μm. (**b, c**) The mRNA expression of GPR81 in HMEC-1 cells co-cultured with indicated treatment. (**d, e**) Verification of GPR81 knockdown efficiency by siRNAs in HMEC-1 cells through qRT-PCR (**d**) and Western blot (**e**). Blots for each antigen were processed in the same experiment in parallel. GPR81 siRNA3 was employed in all the subsequent experiments in Fig. 4. Mean ± SD (n = 6 biologically independent samples/group). The immunofluorescence staining was repeated three times independently with similar results. Analyses: unpaired Student's t-test in **a**; One-way ANOVA with Tukey's post hoc test in **b** and **d**; Two-way ANOVA with Tukey's post hoc test in **c**. The unpaired Student's t-test was conducted as two-sided tests. One or two-way ANOVA tests were performed as two-sided analyses and adjusted for multiple comparisons in the statistical analyses. ns, not significant. HC, healthy control; Pso, psoriasis patients; MFI, mean fluorescence intensity. Source data are provided as a Source Data file.

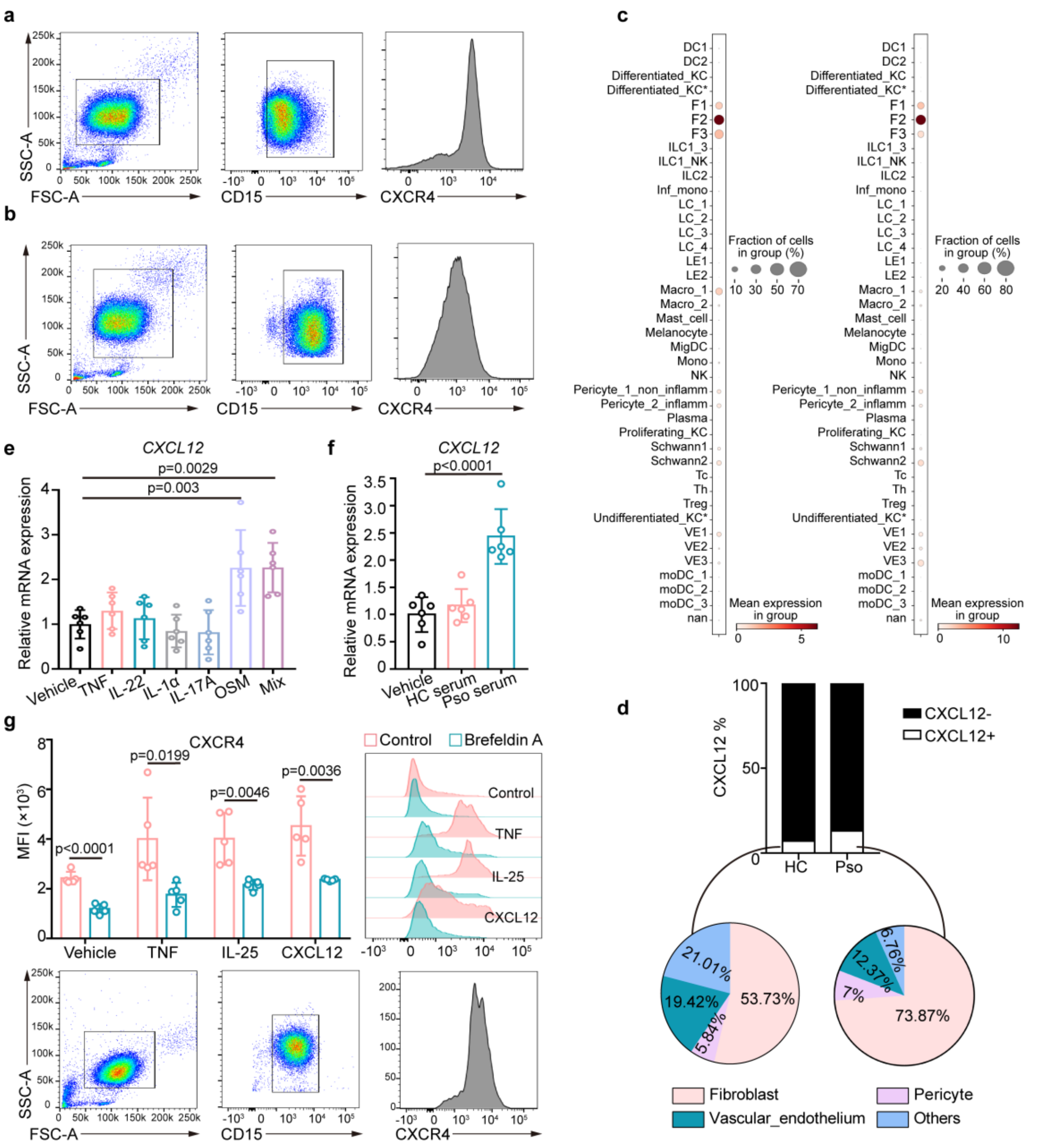

Supplementary Fig. 7. CXCL12 is mainly derived from vascular endothelium and fibroblasts in psoriatic lesions. (**a, b**) Gating strategy for CXCR4 expression on neutrophils, related to Fig. 5a, c. Isolated neutrophils from peripheral blood were used for analysis. (**c, d**) Expressions of CXCL12 in each cluster were analyzed from scRNA-seq analysis (https://developmental.cellatlas.io/diseased-skin) (**c**) and the proportions were determined (**d**). The y-axis represents log-normalized expression. (**e, f**) Expression of CXCL12 in HMEC-1 cells treated with individual pro-inflammatory cytokine/mixture (IL-17A, IL-22, oncostatin M, TNF, and IL-1α) (**e**) and psoriatic serum (**f**). (**g**) Neutrophils isolated from healthy controls were incubated with Brefeldin A for 4 h, followed by treatment with TNF, IL-25, and CXCL12 for 2 h, and CXCR4 MFI was quantified. Mean ± SD (n = 5, 6 biologically independent samples/group). Analyses: one-way ANOVA with Tukey's post hoc test in **e** and **f**; Two-way ANOVA

with Tukey's post hoc test in **g**. One or two-way ANOVA tests were performed as two-sided analyses and adjusted for multiple comparisons in the statistical analyses. ns, not significant. MFI, mean fluorescence intensity; OSM, oncostatin M. Source data are provided as a Source Data file.

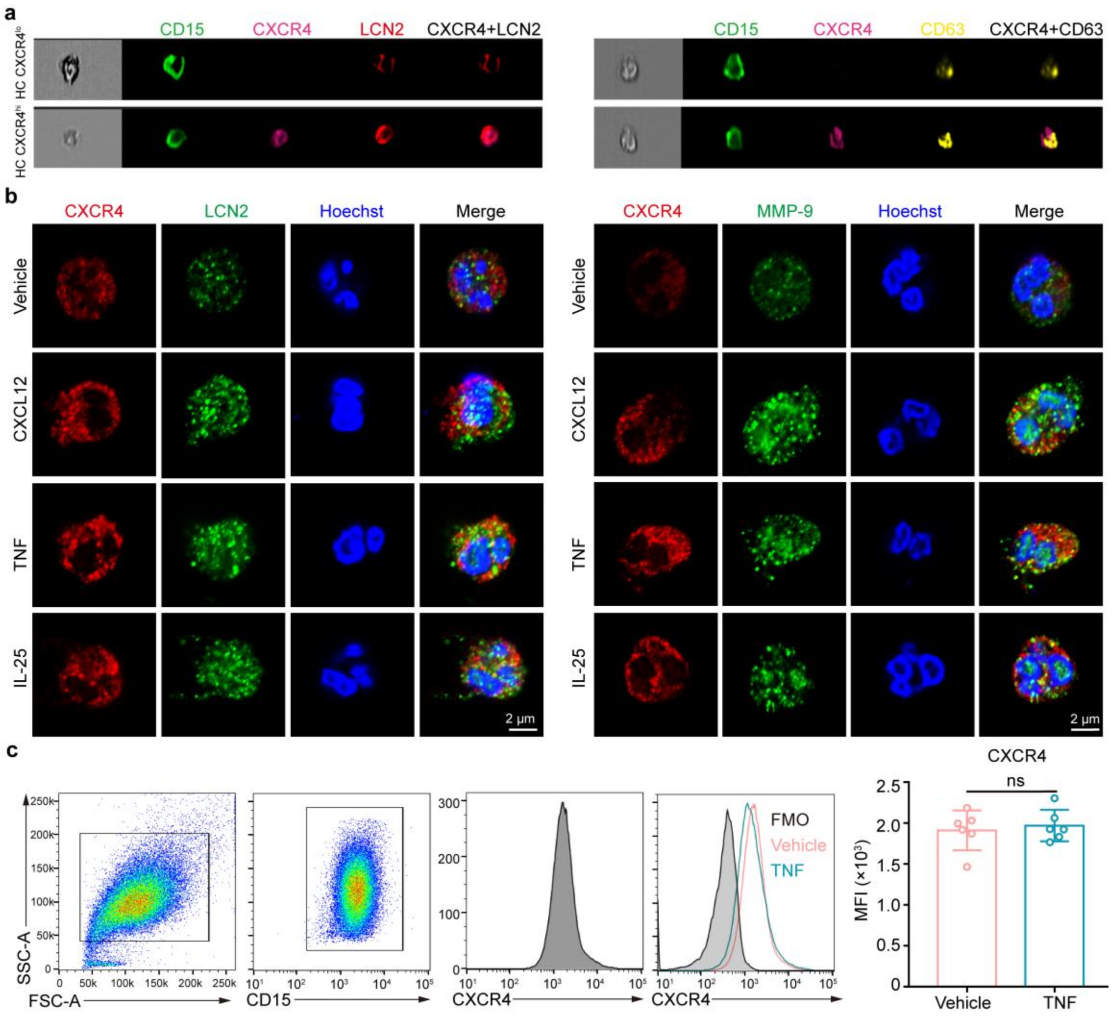

Supplementary Fig. 8. CXCR4 expression on neutrophils is not regulated by degranulation. (**a**) CXCR4 (purple), LCN2 (red), and CD63 (yellow) distribution on CXCR4^{hi} and CXCR4^{lo} neutrophils of healthy controls visualized and quantified by ImageStream analysis. Images are from one representative experiment out of three. The scale bar indicates 7 µm. (**b**) Representative immunofluorescence co-staining of CXCR4 (red) and granule markers in neutrophils treated with TNF, IL-25, and CXCL12. Scale bar = 2 µm. (**c**) Neutrophils were isolated from healthy controls and treated with TNF (100 ng/ml) for 30 min to induce degranulation and the MFI of CXCR4 was quantified. Mean ± SD (n = 6 biologically independent samples/group). The immunofluorescence staining was repeated three times independently with similar results. ns, not significant. Unpaired Student's t-test in **c** was conducted as two-sided tests. FMO, Fluorescence Minus One; MFI, mean fluorescence intensity. Source data are provided as a Source Data file.

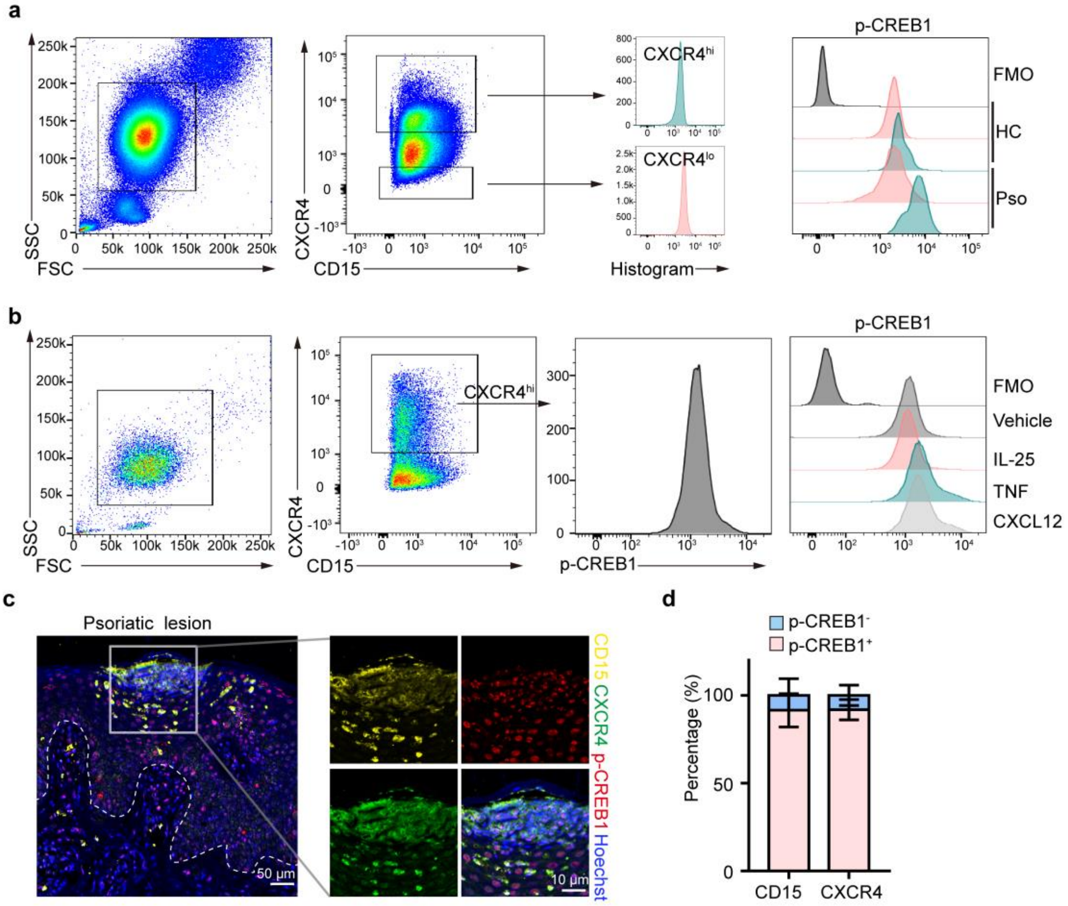

Supplementary Fig. 9. CREB1 promotes development of CXCR4^{hi} neutrophils. (**a**) Gating strategy for p-CREB1 in CXCR4^{lo} and CXCR4^{hi} neutrophils from healthy controls and psoriasis patients, related to Fig. 6c. (**b**) Gating strategy for p-CREB1 in CXCR4^{hi} neutrophils from healthy controls with indicated treatment, related to Fig. 6d. (**c**) Representative immunofluorescence staining of CD15 (yellow), CXCR4 (green), and p-CREB1 (red) in inflamed psoriatic skin (n = 6). Scale bar = 50 µm, 10 µm. **(d)** Quantitation of p-CREB1/CD15 and p-CREB1/CXCR4 double positive cells in psoriatic lesions. Mean ± SD (n = 6 biologically independent samples/group). The immunofluorescence staining was repeated three times independently with similar results. Analyses: one-way ANOVA with Tukey's post hoc test in **d**. FMO, Fluorescence Minus One; HC, healthy control; Pso, psoriasis patients. Source data are provided as a Source Data file.

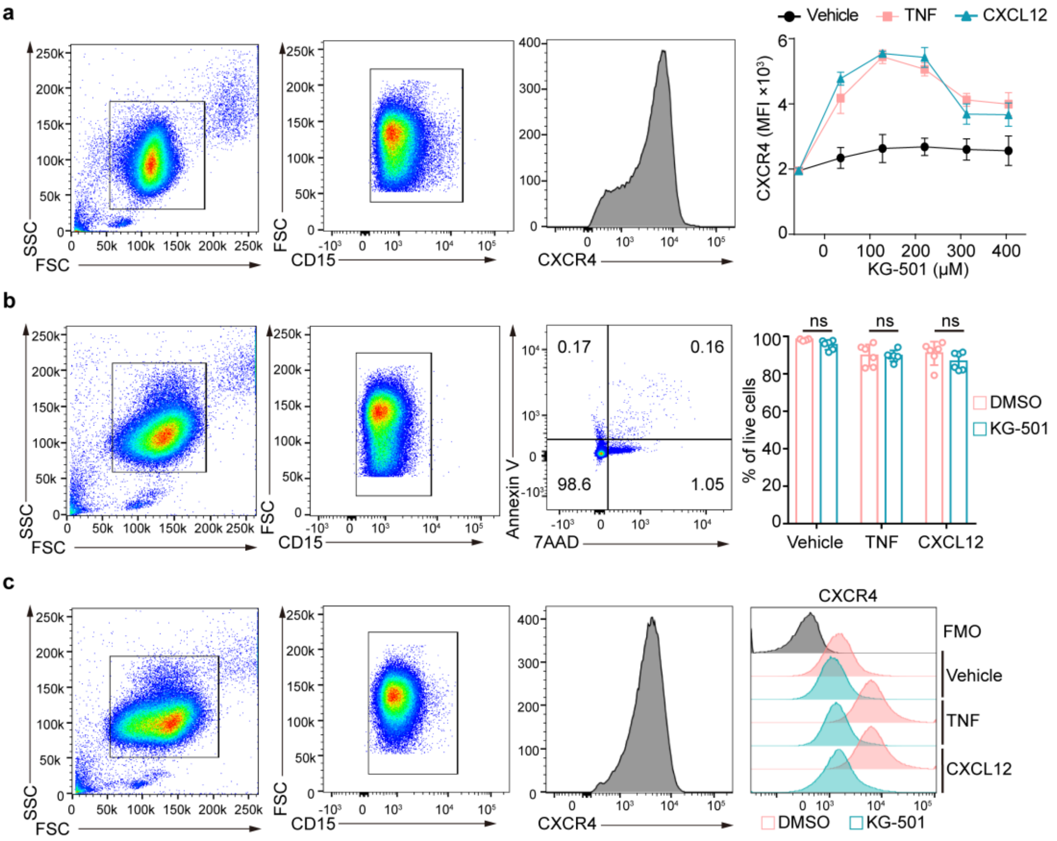

Supplementary Fig. 10. Verification of CREB1 inhibition efficiency. (**a**) Gating strategy and results for CXCR4 expression on neutrophils treated with CREB1 inhibitor (KG-501) at indicated concentrations. (**b**) Neutrophils from healthy controls were pre-treated with CREB1 inhibitor (KG-501, 300 μM) for 1 h, followed by CXCL12 and TNF stimulation for 2 h. The effect of the CREB1 inhibitor (KG-501) on cell viability was analyzed. Bar graph shows the percentage of live cells measured by Annexin V and 7AAD using a flow cytometer. (**c**) Neutrophils from healthy controls were pre-treated with CREB1 inhibitor (KG-501, 300 μM) for 1 h, followed by CXCL12 and TNF stimulation for 2 h. The expression of CXCR4 on neutrophils was analyzed, related to Fig. 6h. Isolated neutrophils from peripheral blood were used. Mean ± SD (n = 6 biologically independent samples/group). One-way ANOVA with Tukey's post hoc test was performed as two-sided analyses and adjusted for multiple comparisons in the statistical analyses. ns, not significant. FMO, Fluorescence Minus One; HC, healthy control; Pso, psoriasis patients. Source data are provided as a Source Data file.

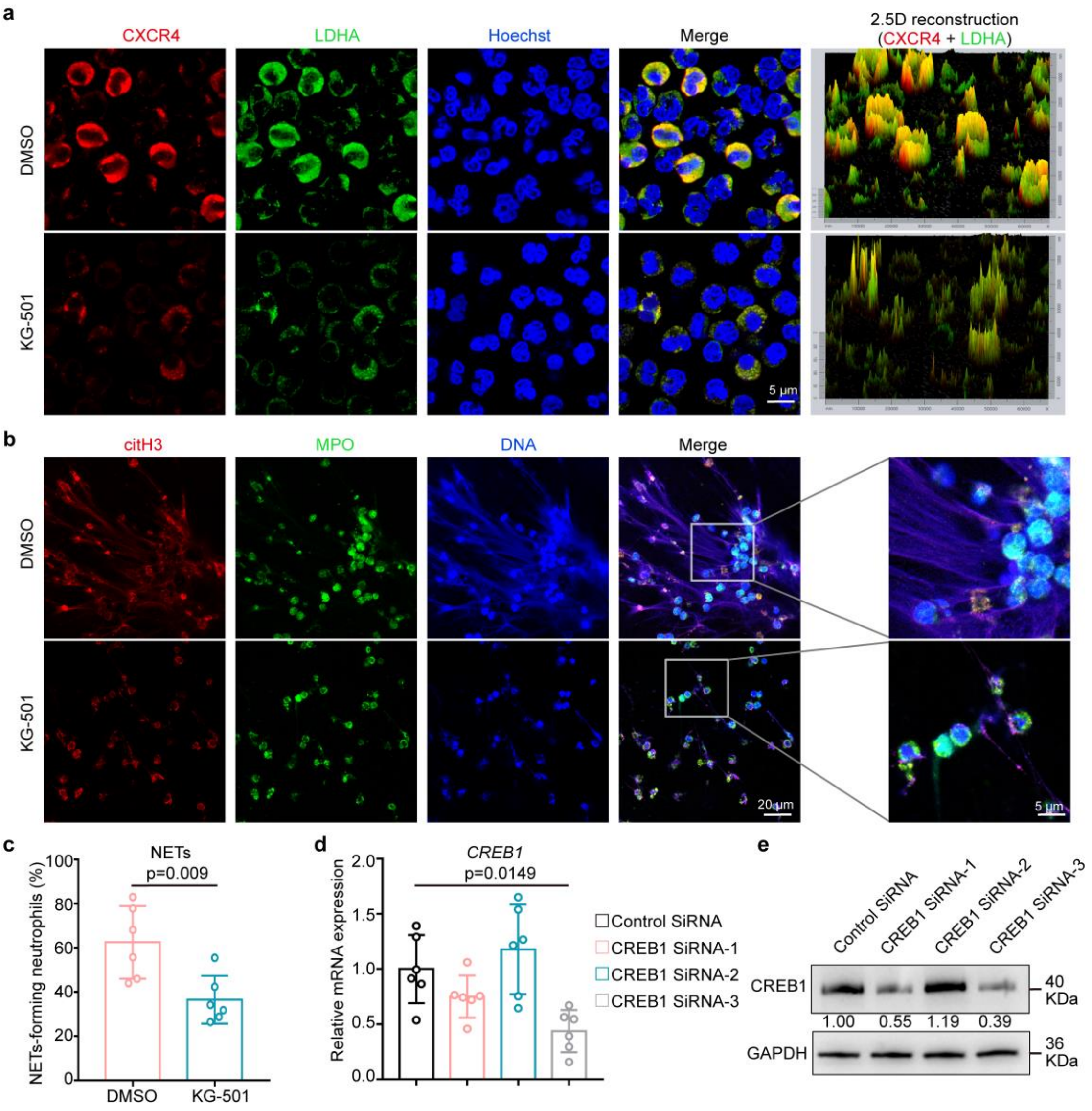

Supplementary Fig. 11. Blocking CREB1 impairs the pro-inflammatory functions of psoriatic CXCR4^{hi} neutrophils. (**a**) Immunofluorescence staining of CXCR4 (red) and LDHA (green) in psoriatic neutrophils *in vitro* with/without KG-501 (300 μM) treatment, and 2.5D reconstruction with overlay was established. Scale bar = 5 μm. (**b, c**) Immunofluorescence staining (red for citH3, green for MPO) and quantification of NETs formation in psoriatic neutrophils. Scale bar = 20 μm, 5 μm. (**d, e**) The efficiency of CREB1 knockdown by siRNAs in dHL-60 cells was verified by qRT-PCR (**d**) and Western blot (**e**). Blots for each antigen were processed in the same experiment in parallel. The CREB1 siRNA3 was employed in all the subsequent experiments. Mean ± SD (n = 6 biologically independent samples/group). The immunofluorescence staining was repeated three times independently with similar results. Analyses: Unpaired Student's t-test in **b;** One-way ANOVA with Tukey's post hoc test in **c**. The unpaired Student's t-test was conducted as two-sided tests. One-way ANOVA test was performed as two-sided analyses and adjusted for multiple comparisons in the statistical

analyses. ns, not significant. citH3, Citrullinated Histone H3; HC, healthy control; MFI, mean fluorescence intensity; NETs; neutrophil extracellular traps; Pso, psoriasis patients. Source data are provided as a Source Data file.

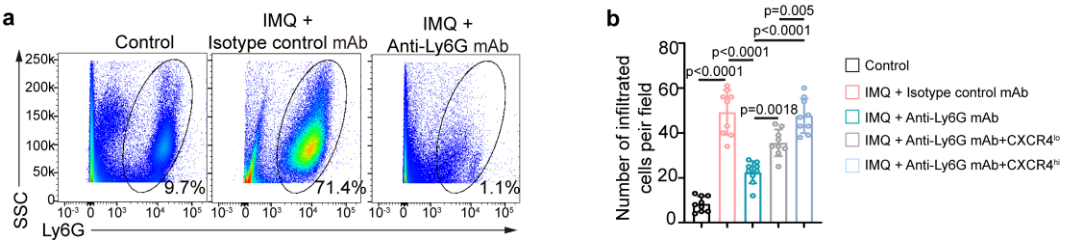

Supplementary Fig. 12. Analysis for the efficiency of neutrophil depletion and infiltration of immune cells in mouse skin, related to Fig. 7. (**a**) Gating strategy for flow cytometric analysis of peripheral neutrophils to validate the efficiency of neutrophil depletion 24 h after injection of anti-Ly6G antibody. (**b**) Infiltrated immune cells were assessed based on H&E staining in Fig. 7a. Mean ± SD (n = 9 vision fields from 6 mice). One-way ANOVA with Tukey's post hoc test was performed as two-sided analyses and adjusted for multiple comparisons in the statistical analyses. IMQ, imiquimod. Source data are provided as a Source Data file.

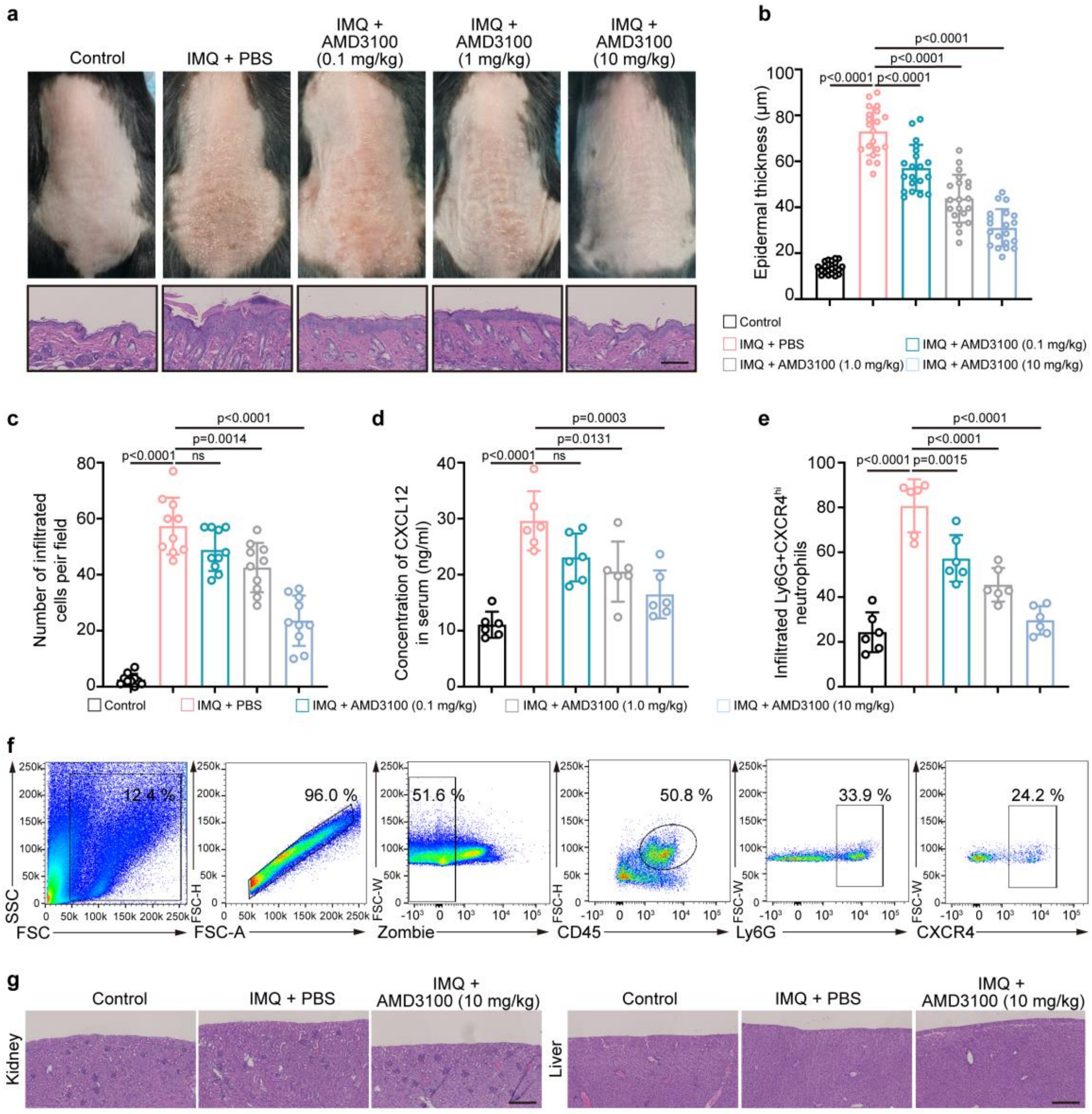

Supplementary Fig. 13. Validation of AMD3100 inhibition efficiency at various concentrations. (**a**) Representative phenotype and H&E staining of IMQ-treated mice in different treatment groups on day 5. Images are representative of six individual mice per group. Control group was topically applied with vaseline cream. Bar = 200 μm. n = 6 mice. (**b, c**) Quantification of epidermis thickness (**b**) and infiltration immune cells (**c**) in the H&E-stained sections. n = vision fields from 6 mice (20 vision fields for **b**, 10 vision fields for **c**). (**d**) Serum level of CXCL12 of IMQ-treated mice in different groups (n = 6 mice). (**e, f**) Infiltrated Ly6G^{+}CXCR4^{hi} neutrophils in inflamed skin (**e**) and the gating strategy for **e** (**f**). n = 6 mice. (**g**) Representative images of H&E for kidney and liver in indicated groups. Bar = 200 μm. n = 6 mice. Mean ± SD. One-way ANOVA with Tukey's post hoc test was performed as two-sided analyses and adjusted for multiple comparisons in the statistical analyses. ns, not significant; IMQ, imiquimod; PBS: phosphate-buffered saline. Source data are provided as a Source Data file.

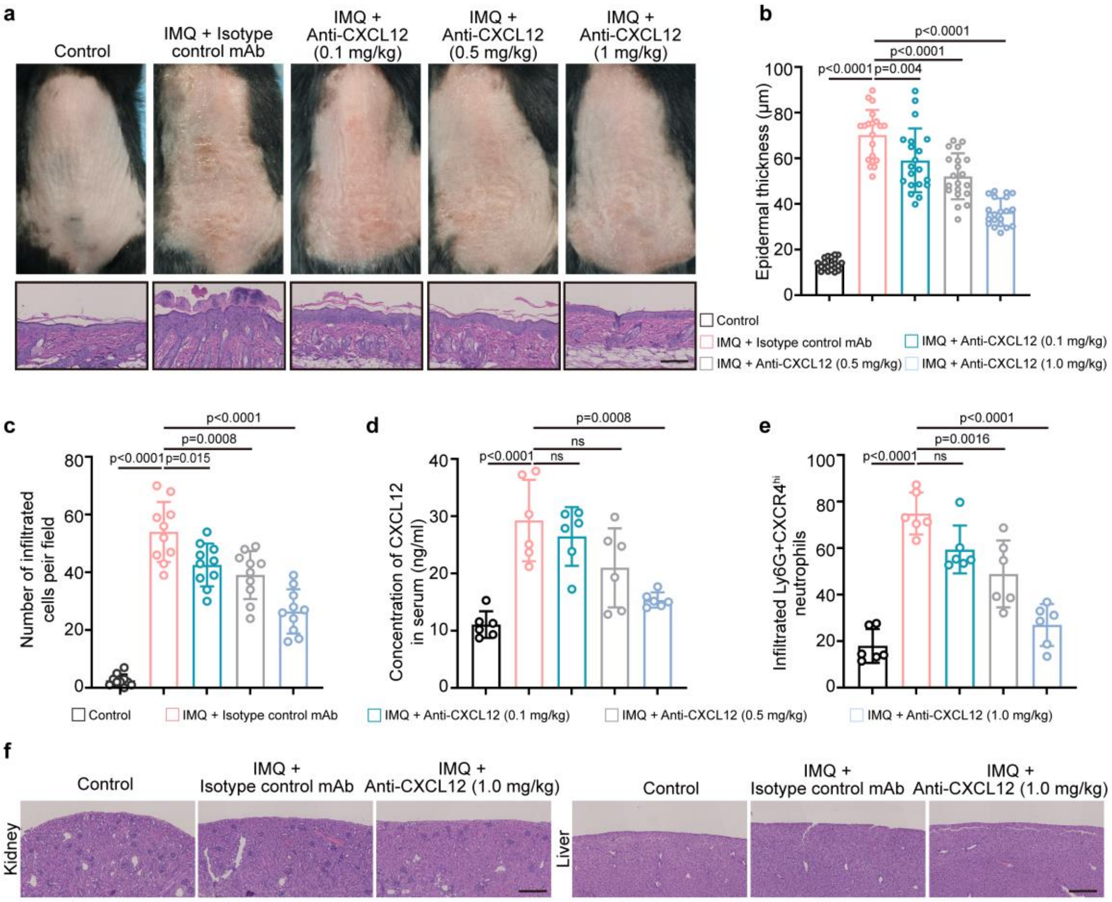

Supplementary Fig. 14. Validation of anti-CXCL12 antibody efficiency at various concentrations. (**a**) Representative phenotype and H&E staining of IMQ-treated mice in different treatment groups on day 5. Images are representative of six individual mice per group. Control group was topically applied with Vaseline cream. Bar = 200 µm. n = 6 mice. (**b, c**) Quantification of epidermis thickness (**b**) and infiltration immune cells (**c**) in the H&E-stained sections. n = vision fields from 6 mice (20 vision fields for **b**, 10 vision fields for **c**). (**d**) Serum level of CXCL12 of IMQ-treated mice in different treatment groups. n = 6 mice. (**e**) Flow cytometric analysis for infiltrated Ly6G^{+}CXCR4^{hi} neutrophils in inflamed skin. n = 6 mice. Gating strategy is the same as Supplementary Fig. 13f. **(f)** Representative images of H&E for kidney and liver in different treatment groups. n = 6 mice. Bar = 200 µm. Mean ± SD. One-way ANOVA with Tukey's post hoc test was performed as two-sided analyses and adjusted for multiple comparisons in the statistical analyses. ns, not significant; IMQ, imiquimod. Source data are provided as a Source Data file.

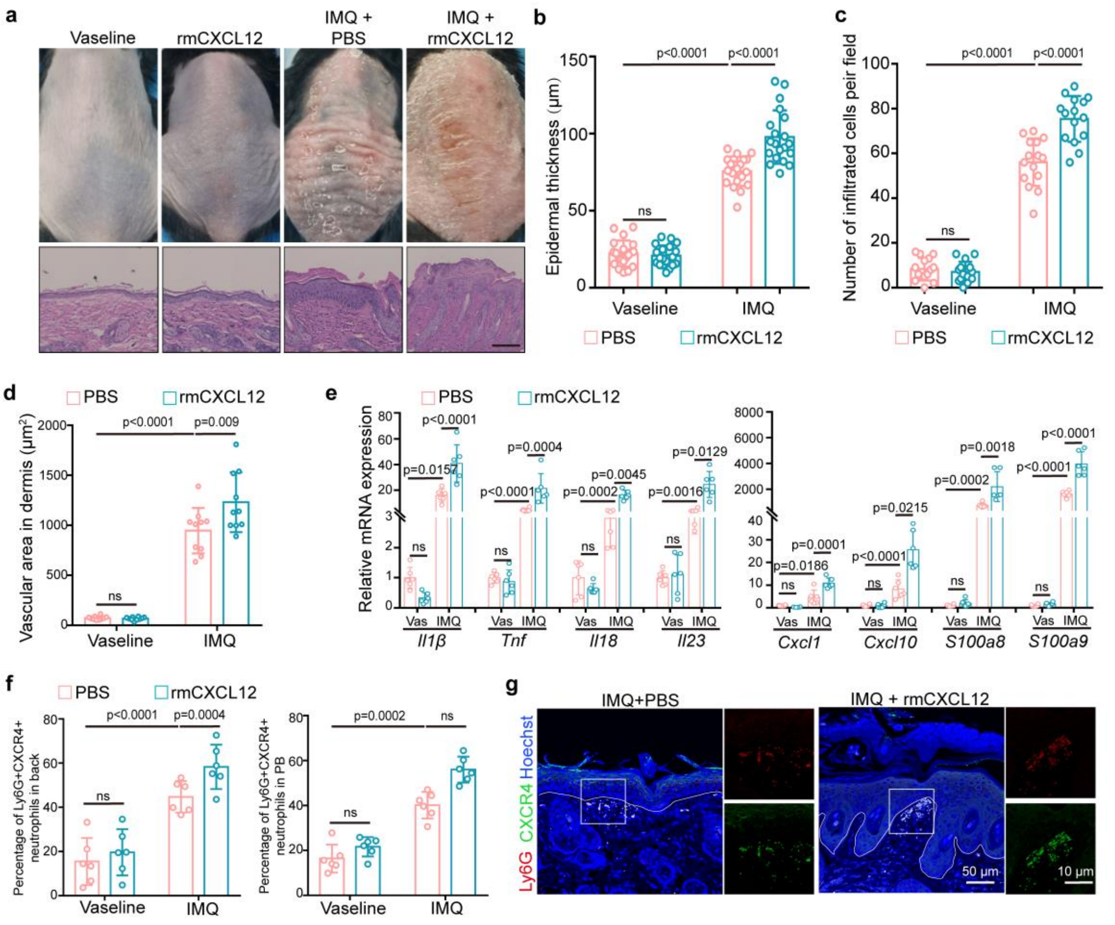

Supplementary Fig. 15. CXCL12 regulates CXCR4^{hi} neutrophils in psoriasis-like mouse model. (**a**) Phenotype and representative H&E staining of IMQ-treated mice in different treatment groups on day 5. Images are representative of six individual mouse per group. Bar = 200 µm. n = 6 mice. (**b-d**) Quantification of epidermis thickness (**b**), infiltration immune cells (**c**), and the dermal vascular area (**d**) in the H&E-stained sections. n = vision fields from 6 mice (20 vision fields for **b**, 15 vision fields for **c**, 10 vision fields for **d**). (**e, f**) Relative mRNA expressions of inflammatory cytokines (**e**) and flow cytometric analysis of infiltrated Ly6G^{+}CXCR4^{hi} neutrophils (**f**) in inflamed skin and peripheral blood. n = 6 mice. (**g**) Representative immunofluorescence staining of Ly6G (red) and CXCR4 (green) in inflamed skin. Scale bar = 50 µm. The result was repeated twice independently from 6 mice with similar results. Data are mean ± SD. Two-way ANOVA with Tukey's post hoc test was performed as two-sided analyses and adjusted for multiple comparisons in the statistical analyses. ns, not significant; IMQ, imiquimod; PB, peripheral blood; rmCXCL12, recombinant murine CXCL12; vas, vaseline. Source data are provided as a Source Data file.

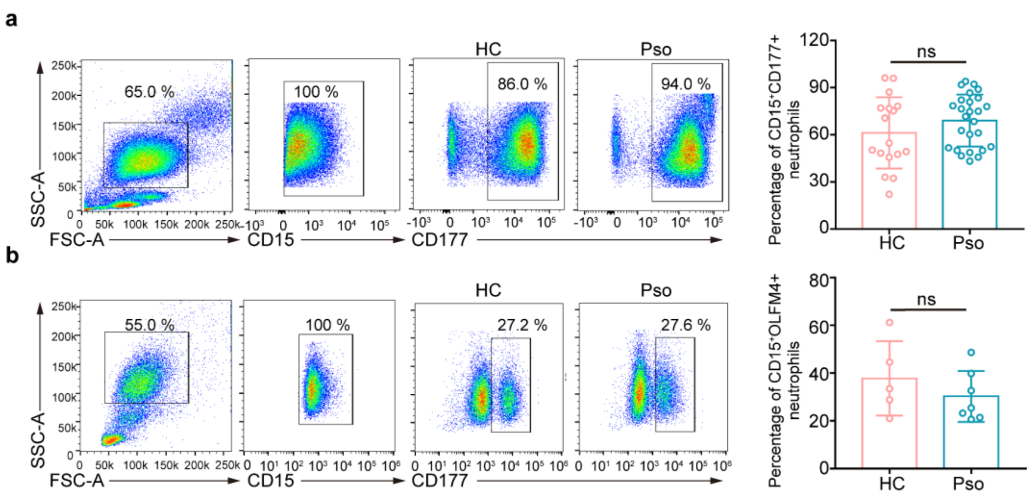

Supplementary Fig. 16. The frequency of CD177$^+$ and OLFM4$^+$ neutrophils in healthy controls and psoriasis patients. (**a-b**) Gating strategy of CD177 or OLFM4 fluorescence intensity on peripheral neutrophils and the proportions of peripheral CD177$^+$ (**a**) or OLFM4$^+$ (**b**) neutrophils from healthy controls and psoriasis patients. n indicates the number of biologically independent samples examined (n = 17 for HC and n = 26 for Pso in **a**, n = 5 for HC and n = 7 for Pso in **b**). Whole blood was used. Data are mean ± SD. Analyses: unpaired Student's t-test. The unpaired Student's t-test was conducted as two-sided tests. ns, not significant. HC, healthy control; Pso, psoriasis patients. Source data are provided as a Source Data file.

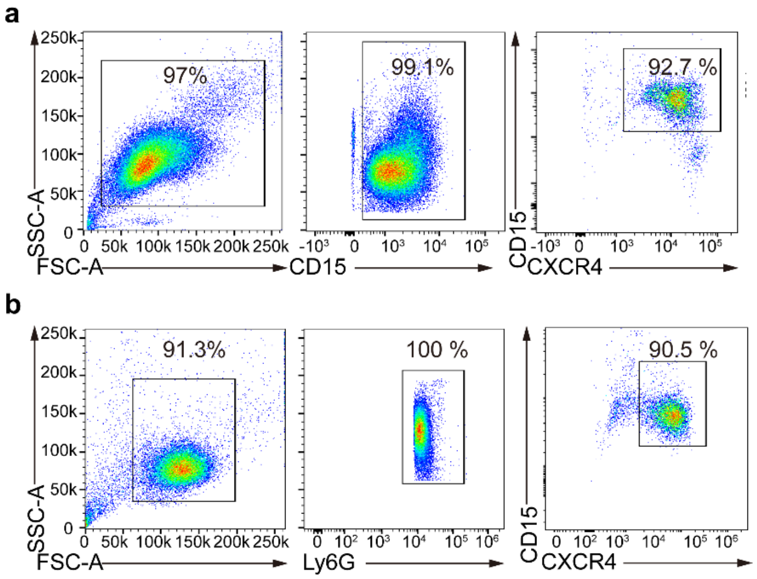

Supplementary Fig. 17. The isolation efficiency of separated CXCR4^{hi} neutrophils via magnetic method. (a, b) The gating strategy and the efficiency of CD15^{+} CXCR4^{hi} (**a**) or Ly6G^{+} CXCR4^{hi} neutrophils (**b**).

Supplementary Table 1. Patient characteristics

Number	Sex	Age (y)	PASI	Disease Duration (y)	Comorbididies
Pso-01	M	23	7.8	2	NO
Pso-02	M	30	6.4	9	NO
Pso-03	F	24	13.8	5	NO
Pso-04	F	33	13	14	NO
Pso-05	F	37	12.3	6	NO
Pso-06	M	38	15.4	13	NO
Pso-07	M	27	19.8	4	NO
Pso-08	M	29	9.1	7	NO
Pso-09	F	36	9	14	NO
Pso-10	F	27	14.2	5	NO
Pso-11	M	35	15.7	8	NO
Pso-12	M	33	7.4	13	NO
Pso-13	F	20	20.1	6	NO
Pso-14	M	43	32.9	3	NO
Pso-15	M	29	12.9	16	NO
Pso-16	F	36	21.9	15	NO
Pso-17	F	47	23.1	4	NO
Pso-18	M	20	10.7	10	NO
Pso-19	M	35	29.4	1	NO
Pso-20	F	21	12	7	NO
Pso-21	M	40	11.4	3	NO
Pso-22	F	32	14.5	13	NO
Pso-23	F	26	17.8	7	NO
Pso-24	M	27	9.6	0.5	NO
Pso-25	F	35	17.5	9	NO
Pso-26	F	33	20.9	8	NO
Pso-27	M	28	16.2	3	NO
Pso-28	F	44	14.5	5	NO
Pso-29	F	26	13.1	4	NO
Pso-30	M	34	16.7	9	NO
Pso-31	F	42	20.5	8	NO
Pso-32	F	29	18.5	4	NO
Pso-33	F	37	17.4	6	NO
Pso-34	M	28	16.5	3	NO
Pso-35	M	44	17.8	4	NO

Supplementary Table 2. Sequences of primers for real time PCR.

Primer		sequences
Mouse	Forward	5'- CAGACTACCTCAACCGTTCCAC -3'
IL-17A	Reverse	5'- TCCAGCTTTCCCTCCGCATTGA -3'
Mouse	Forward	5'- CACACTCACAAACCACCAAGTG-3'
TNF	Reverse	5'- GCAGCCTTGTCCCTTGAAGA-3'
Mouse	Forward	5'- CAAGGAAATCACCATGCCCTCTA-3'
S100A8	Reverse	5'- ACCATCGCAAGGAACTCCTCGA -3'
Mouse	Forward	5'- TGGTGGAAGCACAGTTGGCAAC -3'
S100A9	Reverse	5'- CAGCATCATACACTCCTCAAAGC -3'
Mouse	Forward	5'- GACTGGCATAGTCGGCAATGGA -3'
CXCR4	Reverse	5'- CAAAGAGGAGGTCAGCCACTGA-3'
Mouse	Forward	5'- GGAGGATAGATGTGCTCTGGAAC -3'
CXCL12	Reverse	5'- AGTGAGGATGGAGACCGTGGTG-3'
Mouse	Forward	5'- TGCCACCTTTTGACAGTGATG-3'
IL-1β	Reverse	5'- TGATGTGCTGCTGCGAGATT-3'
Mouse	Forward	5'- TTGACTTGGACCAGCAGGTGTG-3'
IL-36	Reverse	5'- GGGTACTTGCATGGGAGGATAG-3'
Mouse	Forward	5'- GACAGCCTGTGTTCGAGGATATG-3'
IL-18	Reverse	5'- TGTTCTTACAGGAGAGGGTAGAC-3'
Mouse	Forward	5'- CATGCTAGCCTGGAACGCACAT-3'
IL-23	Reverse	5'- ACTGGCTGTTGTCCTTGAGTCC-3'
Mouse	Forward	5'- CATCACTGCCACCCAGAAGACTG-3'
β-actin	Reverse	5'- ATGCCAGTGAGCTTCCCGTTCAG-3'
Mouse	Forward	5'- TCCAGAGCTTGAAGGTGTTGCC -3'
CXCL1	Reverse	5'- AACCAAGGGAGCTTCAGGGTCA-3'
Mouse	Forward	5'- ATCATCCCTGCGAGCCTATCCT-3'
CXCL10	Reverse	5'- GACCTTTTTTGGCTAAACGCTTTC-3'
Human	Forward	5'- CGGACTGTGATGGTCAACCTGA -3'
IL-17A	Reverse	5'- GCACTTTGCCTCCCAGATCACA -3'
Human	Forward	5'- CCTGTGAGGAGGACGAACAT-3'
TNF	Reverse	5'- TTTGAGCCAGAAGAGGTTGAG-3'
Human	Forward	5'- GAGAGTGATTGAGAGTGGACCAC -3'
IL-8	Reverse	5'- CACAACCCTCTGCACCCAGTTT-3'
Human	Forward	5'- GATAGCCAGCCTAGAGGTATGG-3'
IL-18	Reverse	5'- CCTTGATGTTATCAGGAGGATTCA-3'
Human	Forward	5'- GCGAAGAAACTGGGAGAGATGTG-3'

HMGB1	Reverse	5’- GCATCAGGCTTTCCTTTAGCTCG-3’
Human<br>LL-37	Forward<br>Reverse	5’- GACACAGCAGTCACCAGAGGAT-3’<br>5’- TCACAACTGATGTCAAAGGAGCC-3’
Human<br>S100A8	Forward<br>Reverse	5’- ATGCCGTCTACAGGGATGACCT -3’<br>5’- AGAATGAGGAACTCCTGGAAGTTA-3’
Human<br>β-actin	Forward<br>Reverse	5’- GGCTACAGCTTCACCACCAC-3’<br>5’- TGCGCTCAGGAGGAGC-3’
Human<br>S100A9	Forward<br>Reverse	5’- GCACCCAGACACCCTGAACCA-3’<br>5’- TGTGTCCAGGTCCTCCATGATG-3’
Human<br>ICAM-1	Forward<br>Reverse	5’- AGCGGCTGACGTGTGCAGTAAT-3’<br>5’- TCTGAGACCTCTGGCTTCGTCA-3’
Human<br>VCAM-1	Forward<br>Reverse	5’- GATTCTGTGCCCACAGTAAGGC-3’<br>5’- TGGTCACAGAGCCACCTTCTTG-3’
Human<br>ZO-1	Forward<br>Reverse	5’- GTCCAGAATCTCGGAAAAGTGCC -3’<br>5’- CTTTCAGCGCACCATACCAACC -3’
Human<br>VE-Cadherin	Forward<br>Reverse	5’- GAAGCCTCTGATTGGCACAGTG -3’<br>5’- TTTTGTGACTCGGAAGAACTGGC -3’
Human<br>Occludin	Forward<br>Reverse	5’- ATGGCAAAGTGAATGACAAGCGG-3’<br>5’- CTGTAACGAGGCTGCCTGAAGT-3’
Human<br>CREB1	Forward<br>Reverse	5’- GACCACTGATGGACAGCAGATC-3’<br>5’- GAGGATGCCATAACAACTCCAGG-3’
Human<br>CXCL12	Forward<br>Reverse	5’- CTCAACACTCCAAACTGTGCCC-3’<br>5’- CTCCAGGTACTCCTGAATCCAC-3’
Human<br>Hif-1α	Forward<br>Reverse	5’- TATGAGCCAGAAGAACTTTTAGGC-3’<br>5’- CACCTCTTTTGGCAAGCATCCTG-3’
Human<br>HK2	Forward<br>Reverse	5’- TTGACCAGGAGATTGACATGGG-3’<br>5’- CAACCGCATCAGGACCTCA-3’
Human<br>Glut1	Forward<br>Reverse	5’- ATTGGCTCCGGTATCGTCAAC-3’<br>5’- GCTCAGATAGGACATCCAGGGTA-3’
Human<br>LDHA	Forward<br>Reverse	5’- TTGACCTACGTGGCTTGGAAG-3’<br>5’- GGTAACGGAATCGGGCTGAAT-3’
Human<br>GPR81	Forward<br>Reverse	5’- GCCTGCCTTTTCGGACAGACTA-3’<br>5’- ACCACCGTAAGGAACACGATGC-3’
