# Supplementary material for: CREB1-driven CXCR4hi neutrophils promote skin inflammation in mouse models and human patients
Source: Nat Commun. 2023 Sep 22;14:5894. doi: 10.1038/s41467-023-41484-3 (PMC10516899; doi:10.1038/s41467-023-41484-3)
Supplement: Supplementary file 3 — Reporting Summary [file 41467_2023_41484_MOESM3_ESM.pdf]

nature portfolio

Corresponding author(s): Shuai Shao, Gang Wang, Johann E. Gudjonsson

Last updated by author(s): Aug 28, 2023

# Reporting Summary

Nature Portfolio wishes to improve the reproducibility of the work that we publish. This form provides structure for consistency and transparency in reporting. For further information on Nature Portfolio policies, see our Editorial Policies and the Editorial Policy Checklist.

## Statistics

For all statistical analyses, confirm that the following items are present in the figure legend, table legend, main text, or Methods section.

| n/a | Confirmed | |
|---|---|---|
| ☐ | ☒ | The exact sample size ($n$) for each experimental group/condition, given as a discrete number and unit of measurement |
| ☐ | ☒ | A statement on whether measurements were taken from distinct samples or whether the same sample was measured repeatedly |
| ☐ | ☒ | The statistical test(s) used AND whether they are one- or two-sided<br>*Only common tests should be described solely by name; describe more complex techniques in the Methods section.* |
| ☒ | ☐ | A description of all covariates tested |
| ☐ | ☒ | A description of any assumptions or corrections, such as tests of normality and adjustment for multiple comparisons |
| ☐ | ☒ | A full description of the statistical parameters including central tendency (e.g. means) or other basic estimates (e.g. regression coefficient) AND variation (e.g. standard deviation) or associated estimates of uncertainty (e.g. confidence intervals) |
| ☐ | ☒ | For null hypothesis testing, the test statistic (e.g. $F$, $t$, $r$) with confidence intervals, effect sizes, degrees of freedom and $P$ value noted<br>*Give $P$ values as exact values whenever suitable.* |
| ☒ | ☐ | For Bayesian analysis, information on the choice of priors and Markov chain Monte Carlo settings |
| ☒ | ☐ | For hierarchical and complex designs, identification of the appropriate level for tests and full reporting of outcomes |
| ☐ | ☒ | Estimates of effect sizes (e.g. Cohen's $d$, Pearson's $r$), indicating how they were calculated |

*Our web collection on statistics for biologists contains articles on many of the points above.*

## Software and code

Policy information about availability of computer code

| | |
|---|---|
| Data collection | Images for histological analysis were acquired with an Olympus VS120 slide scanner (Olympus Corporation, Tokyo, Japan).<br>Flow cytometry data was collected by FACScan (649225, BD LSRFortessaTM Cell Analyzer).<br>Bound antibodies were examined using the ECL western blotting detection system (Universal Hood II, Bio-Rad).<br>Quantitative real-time PCR was performed with Bio Rad CFX384 PCR System (CFX384 Touch, Bio-Rad).<br>The integrity of RNA was assessed using the Agilent 2100 Bioanalyzer (Agilent Technologies, Santa Clara).<br>Fluorescence spectrophotometry was performed with an excitation wavelength of 494 nm and an emission wavelength of 521 nm (Varioskan LUX 3020-265, Thermo Scientific).<br>Images for immunofluorescence were acquired using a Zeiss LSM 880 confocal microscope (Zeiss Optotechnik, Jena, Germany).<br>Flow imaging was done using Amnis ImageStream markII Imaging Flow Cytometer (Luminex). |
| Data analysis | Flow cytometry data was analyzed with FlowJo software (Tree Star), version 10.8.1.<br>Protein bands were quantified by Image Lab (Bio-Rad Laboratories, Inc.), version 5.2.1.<br>Images for immunofluorescence were analyzed with ZEN software (ver 3.7, Carl Zeiss Microscopy GmbH).<br>Images for histological analysis analyzed by NDP2 viewer software (HAMAMATSU Photonics).<br>GraphPad Prism version 9.5.0 software (GraphPad Software, USA) was used for all data analysis.<br>Flow imaging was analyzed using the IDEAS v 6.2 software (Amnis Corporation). |

For manuscripts utilizing custom algorithms or software that are central to the research but not yet described in published literature, software must be made available to editors and reviewers. We strongly encourage code deposition in a community repository (e.g. GitHub). See the Nature Portfolio guidelines for submitting code & software for further information.

## Data

Policy information about availability of data

All manuscripts must include a data availability statement. This statement should provide the following information, where applicable:

- Accession codes, unique identifiers, or web links for publicly available datasets
- A description of any restrictions on data availability
- For clinical datasets or third party data, please ensure that the statement adheres to our policy

All data generated in this study are available in this manuscript, supplementary information, and Source Data files. A data availability statement is included in the manuscript. All antibody information is provided in the Methods section and Reporting summary. The immunoblotting data generated in this study are provided in the Source Data file. The statistical raw data are provided in the Source data file. Source data are provided with this paper. The raw sequence data generated in this study has been deposited in the Genome Sequence Archive in National Genomics Data Center, Beijing Institute of Genomics (BIG), Chinese Academy of Sciences under accession code HRA005230 [https://ngdc.cncb.ac.cn/search/?dbId=hra&q=HRA005230]. The human publicly available data used in this study are available in the 10X genomics database (https://developmental.cellatlas.io/diseased-skin).

## Human research participants

Policy information about studies involving human research participants and Sex and Gender in Research.

| | |
|---|---|
| Reporting on sex and gender | This study applied in both sexes. Sex and gender were not considered in study design. |
| Population characteristics | The human neutrophils were isolated from peripheral blood of 18-60 years-old psoriasis patients and age- and sex-matched healthy volunteers. |
| Recruitment | Patients enrolled in our study fulfilled the diagnostic criteria for psoriasis and disease activity was scored by the Psoriasis Area Severity Index (PASI). Patients were randomly recruited from outpatient, inpatient and were eligible to participate if they: were ≥18 years of age; regular work schedules and sleep-wake patterns in the preceding 4 weeks; no other autoimmune or systemic diseases and were not receiving systemic treatment in the recent 4 weeks. Controls were collected from sex-, and age-matched healthy volunteers. The patients were recruited according to the objective criteria of age, gender and health status and no self-selection bias affected the recruitment. |
| Ethics oversight | All analyses of human materials were done in full agreement with our institutional guidelines, with the approval of the Ethical committee of the Xijing Hospital, the Fourth Military Medical University (KY20203171-1). Written informed consent was obtained from each participant. |

Note that full information on the approval of the study protocol must also be provided in the manuscript.

# Field-specific reporting

Please select the one below that is the best fit for your research. If you are not sure, read the appropriate sections before making your selection.

☒ Life sciences ☐ Behavioural & social sciences ☐ Ecological, evolutionary & environmental sciences

For a reference copy of the document with all sections, see nature.com/documents/nr-reporting-summary-flat.pdf

# Life sciences study design

All studies must disclose on these points even when the disclosure is negative.

| | |
|---|---|
| Sample size | The minimum of samples in each experiment was n=6, and up to n=54. The exact n for each experiment was described in corresponding figure legends. Sample sizes were determined based on expected effect size and variability within the sample, our previous researches (PMID:31899186, 26979478, 32888954), and standards in the field. Low variability between the same type of samples, indicated as SD, confirming that n=6 samples is sufficient to observe statistically significant differences between relevant groups. Sample size was indicated in each figure legend. In the in vivo experiments, 6 mice/group was sufficient to identify differences between groups with at least 90% power and a 5% significance level. |
| Data exclusions | No data were excluded from the analyses. |
| Replication | At least two or three independent experiments were performed for each experiments, each panel presented the representative data. All reported data were reproduced reliably. |
| Randomization | For in vivo experiments, mice were randomized prior to treatments. For all the other experiments, healthy donors and psoriasis patients were randomly selected in order to avoid potential biases. All of the patients had no other autoimmune or systemic diseases and were not receiving systemic treatments. Controls were collected from sex-, and age-matched healthy volunteers. For cell culture, cells were divided into each plates and assigned into different treatment groups randomly. |

Blinding

For in vivo experiments, researchers were blinded to the treatment each animal received until after data were analyzed. For human samples, when possible, the order of acquiring the sample during flow cytometry was hidden for the investigator during the acquisition of all samples and the gating of results. The the samples were identified into groups at the stage of entering the data into the statistical package. For other experiments, blinding was not possible since the primary investigators performed the experiments from the beginning to the end due to the technical nature of the experiments.

# Reporting for specific materials, systems and methods

We require information from authors about some types of materials, experimental systems and methods used in many studies. Here, indicate whether each material, system or method listed is relevant to your study. If you are not sure if a list item applies to your research, read the appropriate section before selecting a response.

## Materials & experimental systems

| n/a | Involved in the study |
|---|---|
| | x Antibodies |
| | x Eukaryotic cell lines |
| x | Palaeontology and archaeology |
| | x Animals and other organisms |
| x | Clinical data |
| x | Dual use research of concern |

## Methods

| n/a | Involved in the study |
|---|---|
| x | ChIP-seq |
| | x Flow cytometry |
| x | MRI-based neuroimaging |

## Antibodies

Antibodies used

Flow cytometry:
FITC conjugated anti-human CD15 (#301904, 1:100, Clone HI98, BioLegend), PE-Cy7 conjugated anti-human CD15 (#301924, 1:100, Clone HI98, BioLegend), PE conjugated anti-human CXCR4 (#306506, 1:100, Clone 12G5, BioLegend), PE-Cy7 conjugated anti-human CXCR4 (#306514, 1:100, Clone 12G5, BioLegend), PE-Cy5 conjugated anti-human CD62L (#304808, 1:100, Clone DREG-56, BioLegend), PE conjugated anti-human CD11b (#393112, 1:100, Clone LM2, BioLegend), Pacific/Blue conjugated anti-human CD11b (#301315, 1:100, Clone ICRF44, BioLegend), PE anti-human CD44 (#338808, 1:100, Clone BJ18, BioLegend), PerCP/Cy5.5 conjugated anti-human CD101 (#331016, 1:100, Clone BB27, BioLegend), PE-Cy5 anti-human CD10 (#312206, 1:100, Clone HI10a, BioLegend), APC/Cy7 conjugated anti-human CD10 (#312212,1:100, Clone HI10a, BioLegend), anti-HK2 (#209847, Rabbit monoclonal EPR20839, 1:60, Abcam), anti-Glut1 (#115730,1:40, Rabbit monoclonal EPR3915, Abcam), PE-anti-Hif1-α (#359704, Clone 546-16, 1:100, BioLegend), PE Donkey anti-rabbit IgG (#406421, 1:100, Clone Poly4064, BioLegend), PE conjugated anti-human CD63 (#353004, 1:100, Clone H5C6, BioLegend), anti-phospho-CREB1 (#9198S, 1:800, Clone 87G3, CST), PerCP/Cy5.5 anti-mouse CD45 (#103132, 1:100, Clone 30-F11, BioLegend), FITC anti-mouse Ly6G (#127606, 1:100, Clone 1A8, BioLegend), PE anti-mouse CXCR4 (#146506, 1:100, Clone L276F12, BioLegend), Zombie UV™ dye (#423102, 1:500, RUO, BioLegend)

Western Blotting:
Mouse monoclonal to CXCR4 (#60042-1-Ig, 1:1000, Clone 4B5E4, Proteintech), Rabbit mAb to ZO-1 (#96587, 1:1000, polyclonal, Abcam), Rabbit polyclonal to VE-cadherin (#33168, 1:1000, polyclonal, Abcam), Rabbit monoclonal to Occludin (#216327, 1:1000, Clone EPR20992, Abcam), Mouse monoclonal to GAPDH (#60004-1-Ig, 1:5000, Clone 1E6D9, Proteintch), Mouse monoclonal to PADI4 (#128086, 1:1000, Clone OTI4H5, Abcam), Rabbit polyclonal to Histone 3 (#5103, 1:1000, Abcam), Rabbit monoclonal to CREB1 (#9197S, 1:1000, Clone 48H2, CST), Goat polyclonal to GPR81 (#106942, 1:500, Abcam), Rabbit monoclonal to phospho-CREB1 (#9198S, 1:400, Clone 87G3, CST), Mouse monoclonal to CBP (#MA5-13634, 1:1000, Clone NM11, Thermo Fisher Scientific), Mouse monoclonal to MMP-9 (#58803,1:100, Clone 56-2A4, Abcam)

In vivo treatment:
Mouse SDF-1 monoclonal antibody (1 mg/kg, MAB310, Clone 79014, R&D Systems), control mouse IgG (1 mg/kg, MAB002, Clone 11711, R&D Systems), AMD3100 hydrate (10 mg/kg, Sigma-Aldrich), Purified anti-Ly6G antibody (#127649, Clone 1A8, Biolegend, the dose of first injection was 100μg, and the subsequent injection dose was 50μg), isotype control antibody (#400565, Biolegend, the dose of first injection was 100μg, and the subsequent injection dose was 50μg)

Immunofluorescence:
Rat monoclonal to Ly6G (#sc-53515,1:100, Clone RB6-8C5, Santa), Rabbit monoclonal to phospho-CREB1 (#9198S, 1:800, Clone 87G3, CST), Mouse monoclonal to CD15 (#241552, 1:100, Clone 153B, Abcam), Rabbit monoclonal to CD15 (#135377, 1:100, Clone SP159, Abcam), Rabbit monoclonal to CXCR4 (#181020, 1:100, Clone EPUMBR3, Abcam), Mouse monoclonal to CXCR4 (#60042-1-Ig,1:100, Clone 4B5E4, Proteintech), Mouse monoclonal to CD31 (#199012, 1:200, Clone C31.3 + JC/70A, Abcam), Rabbit polyclonal to GPR81 (#PA5-114741, 1:100, Invitrogen), Rabbit monoclonal to Vimentin (#16700, 1:500, Clone SP20, Abcam), Rabbit polyclonal to CXCL12 (#17402-1-AP, 1:100, Proteintech), Rabbit polyclonal to ZO-1 (#96587, 1:100, Abcam), Rabbit polyclonal to VE-cadherin (#33168, 1:100, Abcam), Rabbit monoclonal to Occludin (#216327, 1:100, Clone EPR20992, Abcam), LDHA Rabbit mAb (#3582S,1:200,Clone C4B5, CST), Mouse monoclonal to CXCR4 (#60042-1-1g, 1:100, Clone 4B5E4, Proteintech), Goat Polyclonal to CXCR4 (#GTX21671, 1:100, GeneTex), Mouse monoclonal to LAMP1 (#25630, 1:400, Clone H4A3, Abcam), Mouse monoclonal to CBP (#MA5-13634, 1:500, Clone NM11, Thermo Fisher Scientific), Mouse monoclonal to MMP-9 (#58803,1:100, Clone 56-2A4, Abcam) and Mouse monoclonal to LCN2 (#23477, 1:400, Clone 5G5, Abcam), Rat monoclonal to Ly6G (#sc-53515, 1:100, Clone RB6-8C5, Santa), Rabbit polyclonal to Histone 3 (#5103, 1:200, Clone 2C7, Abcam), Mouse monoclonal to MPO (#25989, 1:100, Abcam), Rabbit monoclonal to LDHA (#52488, 1:200, Clone EP1566Y, Abcam), goat anti-mouse IgG cy3 (#97035, 1:1000, Abcam), goat anti-mouse IgG Alexa Fluor

488 (#150113, 1:1000, Abcam), goat anti-rabbit IgG Alexa Fluor 488 (#150077, 1:1000, Abcam), goat anti-rabbit IgG cy3 (#6939, 1:1000, Abcam).

Imaging Flow Cytometry
Rabbit monoclonal to Lipocalin-2 (#125075, 1:400, Clone EPR5084, abcam), Rabbit monoclonal to MMP9 (#76003, 1:500, Clone EP1254, abcam), APC anti-mouse IgG1 Antibody (#406610, 1:100, Clone RMG1-1, Biolegend), Brilliant Violet 421™ Donkey anti-rabbit IgG (minimal x-reactivity) Antibody (#406410, 1:100, Clone Poly4064, Biolegend)

Validation

All antibodies used in our study are commercially available and validated. Antibodies were all titrated to determine the optimal concentration. Any further informations on the validation performed by the manufacturers can be retrieved at their website. All antibodies were first confirmed with their specific staining using known positive and negative cells with expected pattern.

Flow cytometry:
FITC conjugated anti-human CD15 (#301904, BioLegend): https://www.biolegend.com/en-us/products/fitc-anti-human-cd15-ssea-1-antibody-712?pdf=true&displayInline=true&leftRightMargin=15&topBottomMargin=15&filename=FITC%20anti-human%20CD15%20(SSEA-1)%20Antibody.pdf&v=20230630093043.

PE-Cy7 conjugated anti-human CD15 (#301924, BioLegend): https://www.biolegend.com/en-us/products/pe-cyanine7-anti-human-cd15-ssea-1-antibody-16708?pdf=true&displayInline=true&leftRightMargin=15&topBottomMargin=15&filename=PE/Cyanine7%20anti-human%20CD15%20(SSEA-1)%20Antibody.pdf&v=20230731093045.

PE conjugated anti-human CXCR4 (#306506, BioLegend):https://www.biolegend.com/en-us/products/pe-anti-human-cd184-cxcr4-antibody-542?pdf=true&displayInline=true&leftRightMargin=15&topBottomMargin=15&filename=PE%20anti-human%20CD184%20(CXCR4)%20Antibody.pdf&v=20230628033023.

PE-Cy7 conjugated anti-human CXCR4 (#306514, BioLegend): https://www.biolegend.com/en-us/products/pe-cyanine7-anti-human-cd184-cxcr4-antibody-5709?pdf=true&displayInline=true&leftRightMargin=15&topBottomMargin=15&filename=PE/Cyanine7%20anti-human%20CD184%20(CXCR4)%20Antibody.pdf&v=20230726063409.

PE-Cy5 conjugated anti-human CD62L (#304808, BioLegend): https://www.biolegend.com/en-us/products/pe-cyanine5-anti-human-cd62l-antibody-654?pdf=true&displayInline=true&leftRightMargin=15&topBottomMargin=15&filename=PE/Cyanine5%20anti-human%20CD62L%20Antibody.pdf&v=20230630093043.

PE conjugated anti-human CD11b (#393112, BioLegend): https://www.biolegend.com/en-us/products/pe-anti-human-cd11b-antibody-16303?pdf=true&displayInline=true&leftRightMargin=15&topBottomMargin=15&filename=PE%20anti-human%20CD11b%20Antibody.pdf&v=20230726063409.

Pacific/Blue conjugated anti-human CD11b (#301315, 1:100, BioLegend): https://www.biolegend.com/en-us/products/pacific-blue-anti-human-cd11b-antibody-2852?pdf=true&displayInline=true&leftRightMargin=15&topBottomMargin=15&filename=Pacific%20Blue%E2%84%A2%20anti-human%20CD11b%20Antibody.pdf&v=20230731093045.

PE anti-human CD44 (#338808, 1:100, BioLegend): https://www.biolegend.com/en-us/products/pe-anti-human-cd44-antibody-5745?pdf=true&displayInline=true&leftRightMargin=15&topBottomMargin=15&filename=PE%20anti-human%20CD44%20Antibody.pdf&v=20230114013553.

PerCP/Cy5.5 conjugated anti-human CD101 (#331016, BioLegend): https://www.biolegend.com/en-us/products/percp-cyanine5-5-anti-human-cd101-bb27-antibody-14942?pdf=true&displayInline=true&leftRightMargin=15&topBottomMargin=15&filename=PerCP/Cyanine5.5%20anti-human%20CD101%20(BB27)%20Antibody.pdf&v=20230114013553.

PE-Cy5 anti-human CD10 (#312206, 1:100, BioLegend): https://www.biolegend.com/en-us/products/pe-cyanine5-anti-human-cd10-antibody-2216?pdf=true&displayInline=true&leftRightMargin=15&topBottomMargin=15&filename=PE/Cyanine5%20anti-human%20CD10%20Antibody.pdf&v=20230114013553.

APC/Cy7 conjugated anti-human CD10 (#312212,1:100, BioLegend): https://www.biolegend.com/en-us/products/apc-cyanine7-anti-human-cd10-antibody-4034?pdf=true&displayInline=true&leftRightMargin=15&topBottomMargin=15&filename=APC/Cyanine7%20anti-human%20CD10%20Antibody.pdf&v=20230701123045.

Rabbit Monoclonal to HK2 (#209847, Abcam): https://www.abcam.cn/products/primary-antibodies/hexokinase-ii-antibody-epr20839-ab209847.html.

Rabbit Monoclonal to Glut1 (#115730, Abcam): https://www.abcam.cn/products/primary-antibodies/glucose-transporter-glut1-antibody-epr3915-ab115730.html.

PE-anti-human Hif1-α (#359704, BioLegend): https://www.biolegend.com/en-us/products/pe-anti-human-hif1alpha-antibody-8796?pdf=true&displayInline=true&leftRightMargin=15&topBottomMargin=15&filename=PE%20anti-human%20HIF1%CE%B1%20Antibody.pdf&v=20230726063409

PE Donkey anti-rabbit IgG (#406421, 1:100, BioLegend): https://www.biolegend.com/en-us/products/pe-donkey-anti-rabbit-igg-minimal-x-reactivity-9751?pdf=true&displayInline=true&leftRightMargin=15&topBottomMargin=15&filename=PE%20Donkey%

20anti-rabbit%20IgG%20(minimal%20x-reactivity)%20Antibody.pdf&v=20220118043120

PE conjugated anti-human CD63 (#353004, BioLegend): https://www.biolegend.com/en-us/products/pe-anti-human-cd63-antibody-7433?pdf=true&displayInline=true&leftRightMargin=15&topBottomMargin=15&filename=PE%20anti-human%20CD63%20Antibody.pdf&v=20230714033116

Rabbit Monoclonal to phospho-CREB1 (#9198S, CST): https://www.cellsignal.cn/products/primary-antibodies/phospho-creb-ser133-87g3-rabbit-mab/9198?_=1690871711016&Ntt=9198&tahead=true

PerCP/Cy5.5 anti-mouse CD45 (#103132, BioLegend): https://www.biolegend.com/en-us/products/percp-cyanine5-5-anti-mouse-cd45-antibody-4264?pdf=true&displayInline=true&leftRightMargin=15&topBottomMargin=15&filename=PerCP/Cyanine5.5%20anti-mouse%20CD45%20Antibody.pdf&v=20230114013553

Western Blotting:
Mouse monoclonal to CXCR4 (#60042-1-Ig, Proteintech): https://www.ptglab.co.jp/products/CXCR4-Antibody-60042-1-Ig.htm

Rabbit mAb to ZO-1 (#96587, Abcam): https://www.abcam.com/products/primary-antibodies/zo1-tight-junction-protein-antibody-ab96587.html

Rabbit polyclonal to VE-cadherin (#33168, Abcam): https://www.abcam.com/products/primary-antibodies/ve-cadherin-antibody-intercellular-junction-marker-ab33168.html

Rabbit monoclonal to Occludin (#216327, Abcam): http://cncc.bingj.com/cache.aspx?q=EPR20992&d=4621118798256525&mkt=zh-CN&setlang=zh-CN&w=VQBZ_XPgoM01cXgMSPTxvYTDkF8z0PD0

Mouse monoclonal to GAPDH (#60004-1-Ig, Proteintch): https://www.ptglab.co.jp/products/GAPDH-Antibody-60004-1-Ig.html

Mouse monoclonal to PADI4 (#128086, Abcam): https://www.abcam.com/products/primary-antibodies/padi4--pad4-antibody-oti4h5-ab128086.html

Rabbit polyclonal to Histone 3 (#5103, Abcam): https://www.abcam.com/products/primary-antibodies/histone-h3-citrulline-r2--r8--r17-antibody-ab5103.html

Rabbit monoclonal to CREB1 (#9197S, CST): https://www.cellsignal.com/products/primary-antibodies/creb-48h2-rabbit-mab/9197

Goat polyclonal to GPR81 (#106942, Abcam): https://www.abcam.com/products/primary-antibodies/gpcr-gpr81-antibody-ab106942.html

phospho-CREB1 Rabbit mAb (#9198S, CST): https://www.cellsignal.cn/products/primary-antibodies/phospho-creb-ser133-87g3-rabbit-mab/9198?_=1690871711016&Ntt=9198&tahead=true

Mouse Monoclonal to CBP (#MA5-13634, Thermo Fisher Scientific): https://www.thermofisher.cn/cn/zh/antibody/product/CBP-Antibody-clone-NM11-Monoclonal/MA5-13634

Mouse monoclonal to MMP-9 (#58803, Abcam): https://www.abcam.com/products/primary-antibodies/mmp9-antibody-56-2a4-ab58803.html

FITC anti-mouse Ly6G (#127606, BioLegend): https://www.biolegend.com/en-us/products/fitc-anti-mouse-ly-6g-antibody-4775?pdf=true&displayInline=true&leftRightMargin=15&topBottomMargin=15&filename=FITC%20anti-mouse%20Ly-6G%20Antibody.pdf&v=20220419121729

PE anti-mouse CXCR4 (#146506, BioLegend): https://www.biolegend.com/en-us/products/pe-anti-mouse-cd184-cxcr4-antibody-9057?pdf=true&displayInline=true&leftRightMargin=15&topBottomMargin=15&filename=PE%20anti-mouse%20CD184%20(CXCR4)%20Antibody.pdf&v=20230114013553

Zombie UV™ dye (#423102, BioLegend): https://www.biolegend.com/en-us/products/zombie-aqua-fixable-viability-kit-8444?pdf=true&displayInline=true&leftRightMargin=15&topBottomMargin=15&filename=Zombie%20Aqua%E2%84%A2%20Fixable%20Viability%20Kit.pdf&v=20230525063016

In vivo treatment:
Mouse SDF-1 monoclonal antibody (MAB310; R&D Systems): https://www.rndsystems.com/cn/products/human-mouse-cxcl12-sdf-1-antibody-79014_mab310

control mouse IgG (MAB002; R&D Systems): https://www.rndsystems.com/cn/products/mouse-igg-1-isotype-control_mab002

Purified anti-Ly6G antibody (#127649, Biolegend): http://www.labome.cn/product/BioLegend/127649.html

Immunofluorescence:
Rat monoclonal to Ly6G (#sc-53515, Santa): https://datasheets.scbt.com/sc-53515.pdf

Rabbit monoclonal to phospho-CREB1 (#9198S, CST): https://www.cellsignal.cn/products/primary-antibodies/phospho-creb-

ser133-87g3-rabbit-mab/9198?_=1690871711016&Ntt=9198&tahead=true

Mouse monoclonal to CD15 (#241552, Abcam): https://www.abcam.com/products/primary-antibodies/cd15-antibody-153b-ab241552.html

Rabbit monoclonal to CD15 (#135377, Abcam): https://www.abcam.com/products/primary-antibodies/cd15-antibody-sp159-ab135377.html

Rabbit monoclonal to CXCR4 (#181020, Abcam): https://www.abcam.com/products/primary-antibodies/cxcr4-antibody-epumbr3-ab181020.html

Mouse monoclonal to CXCR4 (60042-1-Ig, Proteintech): https://www.ptgcn.com/products/CXCR4-Antibody-60042-1-Ig.html

Mouse monoclonal to CD31 (#199012, Abcam): https://www.abcam.com/products/primary-antibodies/cd31-antibody-c313--jc70a-ab199012.html

Rabbit polyclonal to GPR81 (#PA5-114741, Invitrogen): https://www.thermofisher.cn/cn/zh/antibody/product/GPR81-Antibody-Polyclonal/PA5-114741

Rabbit monoclonal to Vimentin (#16700, Abcam): https://www.abcam.com/products/primary-antibodies/vimentin-antibody-sp20-ab16700.html

CXCL12 Rabbit mAb (#17402-1-AP, Proteintech): https://www.thermofisher.cn/cn/zh/antibody/product/CXCL12-SDF-1-Antibody-Polyclonal/17402-1-AP

Rabbit polyclonal to ZO-1 (#96587, Abcam): https://www.abcam.cn/products/primary-antibodies/zo1-tight-junction-protein-antibody-ab96587.html

Rabbit polyclonal to VE-cadherin (#33168, Abcam): https://www.abcam.cn/products/primary-antibodies/ve-cadherin-antibody-intercellular-junction-marker-ab33168.html

Rabbit monoclonal to Occludin (#216327, Abcam): https://www.abcam.cn/products/primary-antibodies/occludin-antibody-epr20992-ab216327.html

Rabbit monoclonal to LDHA (#3582S, CST): https://www.cellsignal.cn/products/primary-antibodies/ldha-c4b5-rabbit-mab/3582

Mouse monoclonal to CXCR4 (#60042-1-1g, Proteintech): https://www.ptgcn.com/Products/CXCR4-Antibody-60042-1-Ig.htm

Goat Polyclonal to CXCR4 (#GTX21671, GeneTex): https://www.genetex.cn/PDF/Download?catno=GTX21671

Mouse monoclonal to LAMP1 (#25630, Abcam): https://www.abcam.cn/products/primary-antibodies/lamp1-antibody-h4a3-ab25630.html

Mouse monoclonal to CBP (#MA5-13634, Thermo Fisher Scientific): https://www.thermofisher.cn/cn/zh/antibody/product/CBP-Antibody-clone-NM11-Monoclonal/MA5-13634

Mouse monoclonal to MMP-9 (#58803, Abcam): https://www.abcam.cn/products/primary-antibodies/mmp9-antibody-56-2a4-ab58803.html

Mouse monoclonal to LCN2 (#23477, Abcam): https://www.abcam.cn/products/primary-antibodies/lipocalin-2--ngal-antibody-5g5-ab23477.html

Rat monoclonal to Ly6G (#sc-53515, Santa): https://www.scbt.com/p/ly-6g-antibody-rb6-8c5/

Rabbit polyclonal to Histone 3 (#5103, Abcam): https://www.abcam.cn/products/primary-antibodies/histone-h3-citrulline-r2--r8--r17-antibody-ab5103.html

Mouse monoclonal to MPO (#25989, Abcam): https://www.abcam.cn/products/primary-antibodies/myeloperoxidase-antibody-2c7-ab25989.html

Rabbit monoclonal to LDHA (#52488 Abcam): https://www.abcam.cn/products/primary-antibodies/lactate-dehydrogenase-antibody-ep1566y-ab52488.html

goat anti-mouse IgG cy3 (#97035, Abcam): https://www.abcam.cn/products/secondary-antibodies/goat-mouse-igg-hl-cy3--preadsorbed-ab97035.html

goat anti-mouse IgG Alexa Fluor 488 (#150113, Abcam): https://www.abcam.cn/products/secondary-antibodies/goat-mouse-igg-hl-alexa-fluor-488-ab150113.html

goat anti-rabbit IgG Alexa Fluor 488 (#150077, Abcam): https://www.abcam.cn/products/secondary-antibodies/goat-rabbit-igg-hl-

alexa-fluor-488-ab150077.html

goat anti-rabbit IgG cy3 (#6939, Abcam): https://www.abcam.cn/products/secondary-antibodies/goat-rabbit-igg-hl-cy3--preadsorbed-ab6939.html

Imaging Flow Cytometry
Rabbit monoclonal to Lipocalin-2 (#125075, abcam): https://www.abcam.cn/products/primary-antibodies/lipocalin-2--ngal-antibody-epr5084-ab125075.html

Rabbit monoclonal to MMP9 (#76003, abcam): https://www.abcam.cn/products/primary-antibodies/mmp9-antibody-ep1254-ab76003.html

APC anti-mouse IgG1 Antibody (#406610, Biolegend): https://www.biolegend.com/en-us/products/apc-anti-mouse-igg1-7022?GroupID=BLG3729

Brilliant Violet 421™ Donkey anti-rabbit IgG (minimal x-reactivity) Antibody (#406410, 1:100, Biolegend): https://www.biolegend.com/en-us/products/brilliant-violet-421-donkey-anti-rabbit-igg-minimal-x-reactivity-7262

# Eukaryotic cell lines

Policy information about cell lines and Sex and Gender in Research

| | |
|---|---|
| Cell line source(s) | Human microvascular endothelial cell line (HMEC-1 cell) were purchased from American Type Culture Collection (ATCC) and cultured as required. HL-60 cell lines were obtained from Procell (CL-0110, Wuhan, China) and cultured as required. |
| Authentication | Morphology analysis with microscopy was used for cell line authentication. |
| Mycoplasma contamination | All cell lines were tested negative for mycoplasma contamination. |
| Commonly misidentified lines (See ICLAC register) | No commonly misidentified line was used in this study. |

# Animals and other research organisms

Policy information about studies involving animals; ARRIVE guidelines recommended for reporting animal research, and Sex and Gender in Research

| | |
|---|---|
| Laboratory animals | The C57BL/6J mice (8-10 weeks old) were purchased from Department of Laboratory Animal Medicine of the Fourth Military Medical University. Mice were randomly assigned to groups of 3-5 mice, then bred and maintained in a specific pathogen-free barrier facility. All animal experiments were approved by the Institutional Animal Care and Use Committee of the Fourth Military Medical University. |
| Wild animals | No wild animals were used in this study. |
| Reporting on sex | There was no sex biased in the animal studies used in this study. |
| Field-collected samples | No filed-collected samples were used in this study. |
| Ethics oversight | All animal experiments were approved by the Institutional Animal Care and Use Committee of the Fourth Military Medical University. |

Note that full information on the approval of the study protocol must also be provided in the manuscript.

# Flow Cytometry

## Plots

Confirm that:

- [x] The axis labels state the marker and fluorochrome used (e.g. CD4-FITC).
- [x] The axis scales are clearly visible. Include numbers along axes only for bottom left plot of group (a 'group' is an analysis of identical markers).
- [x] All plots are contour plots with outliers or pseudocolor plots.
- [x] A numerical value for number of cells or percentage (with statistics) is provided.

## Methodology

| | |
|---|---|
| Sample preparation | For the analysis of neutrophil phenotypes and functions, total blood leukocytes were collected from sex-, and age-matched |

healthy controls and psoriasis patients. Peripheral blood was collected by anticoagulant tubes and all the subsequent experiments were performed immediately after blood collection. 7 ml blood was layered on top of 7 ml of PolymorphprepTM (1114683, Axis-Shield, Norway) in a 15 mL centrifugation tube. The tube was centrifuged at 350 g at 20 °C for 30 minutes. The polymorphonuclear cell layer was collected and red blood cells were removed using Red Blood Lysing Buffer (FXP001, 4A Biotech Co., Ltd, Beijing, China). Freshly isolated neutrophils were suspended in PBS.

For the analysis of mouse skin, 1cm × 1cm dorsal skin was cut off and transferred to an EP tube containing 1mL Hank's Balanced Saline Solution (HBSS, H4641, Sigma). The skin was washed rigorously by quickly shaking up and down by hand for 15 s × 3 times. The skin was cut into pieces (< 0.5 mm in size) in a 6-well plate placed on ice with dulbecco's modified eagle medium (DMEM, 11885-084, Gibco) (not supplemented with FBS) containing 1 mg/mL Collagenase P (11213857001, Roche) and 0.2 mg/mL DNase I (AMPD1, Sigma). The samples were incubated in a 37 °C cell culture incubator for 60 min and pipetted every 20 min to gently mix the cells. Cell suspensions were filtered through 40 µm cell strainer.

Instrument

BD LSR Fortessa, ImageStream Mark II

Software

The samples were collected by FACScan (649225, BD LSRFortessaTM Cell Analyzer).

Data were analyzed with Flowjo v10 (Tree Star) and plotted using GraphPad Prism 8.0.

For imaging flow cytometry, approximately 1-2x10^4 cells were collected from each sample and data were analyzed using image analysis software (IDEAS 6.2; Amnis Corp)

Cell population abundance

The abundance is depended on the specific population.

Gating strategy

Debris was removed by gating on the main cell population using the FSC/SSC gating. Different gating strategies for the isolation of neutrophils are indicated in Figure legends and Supplementary Information.

For imaging flow cytometry, single stained control cells were used to compensate fluorescence between channel images to avoid emission spectra overlap. Cells were gated for single cells with the area and aspect ratio features and for focused cells, using the Gradient RMS feature. Cells were then gated for the selection of positively stained cells based on their pixel intensity.

☒ Tick this box to confirm that a figure exemplifying the gating strategy is provided in the Supplementary Information.
